# Toward Functional Synthetic Cells: In‐Depth Study of Nanoparticle and Enzyme Diffusion through a Cross‐Linked Polymersome Membrane

**DOI:** 10.1002/advs.201801299

**Published:** 2019-01-11

**Authors:** Hannes Gumz, Susanne Boye, Banu Iyisan, Vera Krönert, Petr Formanek, Brigitte Voit, Albena Lederer, Dietmar Appelhans

**Affiliations:** ^1^ Leibniz‐Institut für Polymerforschung Dresden e.V. Hohe Straße 6 01069 Dresden Germany; ^2^ School of Science Faculty of Chemistry and Food Chemistry Technische Universität Dresden 01062 Dresden Germany; ^3^ Cluster of Excellence “Center for Advancing Electronics Dresden” Technische Universität Dresden 01062 Dresden Germany; ^4^ Max‐Planck‐Institute for Polymer Research Ackermannweg 10 55128 Mainz Germany

**Keywords:** cell‐like uptake functions, enzymes, membrane diffusion, polymeric vesicles, postloading

## Abstract

Understanding the diffusion of nanoparticles through permeable membranes in cell mimics paves the way for the construction of more sophisticated synthetic protocells with control over the exchange of nanoparticles or biomacromolecules between different compartments. Nanoparticles postloading by swollen pH switchable polymersomes is investigated and nanoparticles locations at or within polymersome membrane and polymersome lumen are precisely determined. Validation of transmembrane diffusion properties is performed based on nanoparticles of different origin—gold, glycopolymer protein mimics, and the enzymes myoglobin and esterase—with dimensions between 5 and 15 nm. This process is compared with the in situ loading of nanoparticles during polymersome formation and analyzed by advanced multiple‐detector asymmetrical flow field‐flow fractionation (AF4). These experiments are supported by complementary i) release studies of protein mimics from polymersomes, ii) stability and cyclic pH switches test for in polymersome encapsulated myoglobin, and iii) cryogenic transmission electron microscopy studies on nanoparticles loaded polymersomes. Different locations (e.g., membrane and/or lumen) are identified for the uptake of each protein. The protein locations are extracted from the increasing scaling parameters and the decreasing apparent density of enzyme‐containing polymersomes as determined by AF4. Postloading demonstrates to be a valuable tool for the implementation of cell‐like functions in polymersomes.

## 1 Introduction

Cells are considered the fundamental building blocks of life. Therefore, substantial efforts have been—and still are—directed toward artificial construction of rudimental cells, protocells, or the so‐called cell mimics.^1^, ^2^, ^3^, ^4^, ^5^, ^6^, ^7^, ^8^, ^9^ Very often these approaches are focused, for example, on mimicking spatially separated biological pathways^8^, ^9^, ^10^, ^11^, ^12^ or on stimulating the even more dynamic processes of fusion in synthetic protocell communities.^13^, ^14^ There is a significant progress in the development of different types of vesicles (e.g., liposomes, polymersomes, hollow capsules, proteinosomes) and their multicompartments to establish cell‐like functions. It is, however, still a challenge to enhance the communication between and within (multi)compartmentalized cell mimics (= protocells). This requires the control over the postloading and exchange of nanometer‐sized biologically active particles (enzyme, proteins, protein complexes, etc.).

### 1.1. Different Restrictions of Membrane Permeability Can be Encountered When Cell Mimics Are Based on Polymeric Vesicles

The usage of even more stable and multiresponsive polymeric vesicles (= polymersomes)^15^ instead of their natural counterpart, the liposomes (consisting of phospholipids),^16^ in the field of cell mimics enables researchers to draw advantage from the plethora of chemical functionalities and composition possibilities of amphiphilic block copolymers.^17^, ^18^, ^19^, ^20^, ^21^, ^22^, ^23^, ^24^, ^25^, ^26^, ^27^, ^28^, ^29^ Yet, the thicker polymersome membrane leads to a significantly decreased permeability compared to liposomes.^17^, ^30^ To solve this problem, membrane proteins have been incorporated into the polymersomes.^31^ These biopores/channels enable the construction of highly permeable membranes with an excellent selectivity, however, only for the exchange of small molecules or ions between polymersomes and their environment.^32^, ^33^ Thus, possibly interesting enzymes and nanoparticles are simply too large to diffuse through the narrow channels. Furthermore, biopores/channels are able to induce only a passive membrane transport,^31^, ^33^ apart from one very recent achievement in integrating pH‐responsive biovalves in a polymersome membrane.^34^ As a consequence, alternative approaches for the membrane transport of nanometer‐sized biomacromolecules (enzymes, proteins, protein complexes, etc.) have to be found for establishing cell‐like communication functions.

In contrast to the exchange of small molecules by membrane proteins^31^ as well as by inherently permeable and passive polymersomes,^8^, ^35^, ^36^ only few reports exist on polymeric vesicles mimicking cell‐like uptake functions for nanoparticles or larger biomacromolecules. For example, the permeability of larger nanometer‐sized particles is triggered by a randomly passive membrane diffusion process^37^ or by a sequential approach using morphological transformation of the entire vesicles followed by solvent exchange or temperature stimulus.^38^, ^39^ Our study will present an alternative way to overcome the limited permeability of cell mimics in the postloading process of nanoparticles (*∅* ≥ 4 nm).

In order to understand the postloading of biomacromolecules by polymersomes as cellular mimics, we introduced the concept of pH switchable, photocross‐linked polymersomes to control their permeability for drug release and enzymatic activity by a pH stimulus.^24^ Cross‐linking avoids the typical disassembly of the polymer membrane upon the application of pH stimulus.^24^, ^40^, ^41^ Moreover, cross‐linked polymersomes exhibit enhanced mechanical stability^24^ and potentially endless switching on and off membrane permeability by pH cycles.^24^, ^42^

**Figure 1:**
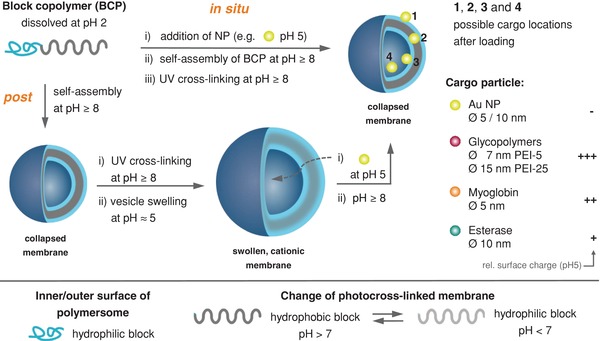
Schematic representation of the in situ loading and postloading strategy and simplified visualization of possible locations of encapsulated cargo within the vesicle membrane: outer (1) and inner (3) hydrophilic surface (= shell), hydrophobic membrane (2), and lumen (4). Key characteristics of cargo nanoparticles (right). Hydrophobic membrane with tertiary amino groups and randomly distributed photocross‐linkable units for the realization of pH‐switchable polymersomes. NP = Nanoparticle.

For mimicking cell functions, e.g., to exchange (macro)molecules as cargo by crossing stimulated membranes, not only the controlled release of cargo from artificial organelles/cells is of interest, but also the uptake procedure of cargo by polymersomes is of importance. In general, it is observed that polymersomes can encapsulate hydrophilic molecules in their lumen, whereas hydrophobic cargo can be loaded into the membrane.^17^, ^18^ This simple model works sufficiently well for the use of small molecules like drugs and dyes, but lacks any deeper consideration of the interactions for larger, nanometer‐sized cargo with the inner and outer surfaces of the polymersomes as well as within the polymersome membrane. In this context, our photocross‐linked polymersomes with reversibly pH switchable membrane permeability through photocross‐linked membrane of polymersome (**Figure** **1**) (for details of polymersome composition, see the Supporting Information) offer the chance to study the postloading of nanometer sized cargo in addition to the frequently used in situ loading. In situ loading describes encapsulation of cargo during the formation of vesicles, whereas postloading refers to cargo encapsulation after the polymersome formation process. For postencapsulation, the polymersome membrane is adjusted at pH ≤ 6.5 in its swollen state when its permeability is highest allowing diffusion of larger particles into the polymersome lumen. At larger pH values (>7), the membrane is collapsed and its permeability is negligible for any organic, hybrid, or macromolecular compounds. Thus, we aim at clarifying the underlying principles which govern the diffusion of nanoparticles through the membrane of our pH switchable polymersomes in a postloading process (Figure 1).

In course of this study, in‐depth characterization of the nanoparticles loading is essential to determine their locations within the polymersomes. Previously, the characterization of polymersome scaffold and size has been carried out by different analytical tools (small angle neutron or X‐ray scattering, cryogenic transmission electron microscopy (cryo TEM), static and dynamic light scattering (DLS)).^43^ However, access to full information on the loaded polymersomes, including membrane composition and density of polymersomes, is thoroughly facilitated by multiple‐detector asymmetrical flow field flow fractionation (AF4) but so far not fully exploited to investigate liposomes^44^, ^45^, ^46^ and polymersomes.^47^, ^48^ Most reports focused only on their size distribution^25^, ^48^, ^49^ or encapsulation efficiency.^38^, ^50^ Here, we show that this highly sophisticated analytical tool enables us to quantify cargo loading and simultaneously to investigate a multitude of polymersome structural characteristics, e.g., size, mass, shape, and density. In contrast to other more common separation methods like liquid chromatography, AF4 does not require a stationary phase,^51^ thus, sample impairment or damage derived from column interactions or strong shear forces can be avoided. In this manner, it was possible for the first time to analyze in detail the effect of in situ loading and postloading method on the membrane properties, thus providing further insight into diffusion and adsorption processes across and into membranes of polymersomes and encapsulation of nanoparticles in polymersome lumen.

Using our established polymersomes with pH switchable cross‐linked membrane, we proved the potential and the limits of postloading of swollen, permeable polymersomes with a variety of cargo nanoparticles/macromolecules with varying dimension and surface charge. Furthermore, the exact/main location of the nanoparticles in the polymersome, e.g., into the lumen or stuck or adsorbed onto the inner and outer sides of the vesicle membrane, should provide significant insight into the parameters which govern the diffusion of the various nanoparticles (Figure 1). For analyzing the effects of particle loading on the integrity of the membrane and the conformation of the vesicles depending on cargo size and loading strategy, gold nanoparticles of two different sizes (5 and 10 nm), glycopolymers (7 and 15 nm), and proteins (5 and 10 nm) were compared (Figure S1 and Table S1, Supporting Information). Finally, the uptake efficiency of enzymes for the fabrication of reversibly switchable enzymatic nanoreactors was evaluated, comparing postloading and in situ loading approach.

## 2 Results and Discussion

### 2.1 In Situ and Post Encapsulation of Gold Nanoparticles

The most distinct proof for cargo encapsulation is to directly visualize its location in the polymersome.^52^, ^53^, ^54^ For this reason gold nanoparticles (AuNPs) of either 5 or 10 nm in diameter were chosen as a cargo, because they offer an excellent contrast for cryo‐TEM measurements.

**Figure 2:**
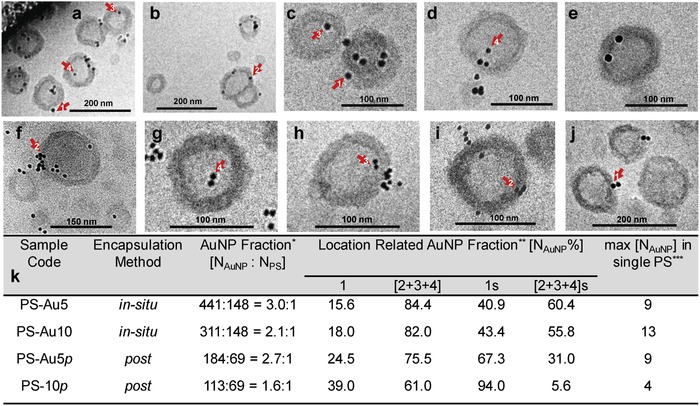
Cryo‐TEM micrographs of AuNP encapsulated polymersomes (PS) at pH 8: a,b) PS‐Au5, c–f) PS‐Au10, g–i) PS‐Au5p, and j) PS‐Au10p. k) Sample code for the fraction of gold nanoparticles within the polymersomes after in situ loading (PS‐Au5 for AuNP with diameter of 5 nm and PS‐Au10 for AuNP with diameter of 10 nm) or postloading (PS‐Au5p for AuNP with diameter of 5 nm and PS‐Au10p for AuNP with diameter of 10 nm) process determined from several cryo‐TEM micrographs. *N_AuNP_ and *N*_PS_ represent the total number of gold nanoparticles integrated with total number of polymersomes, respectively. **NAuNP% is the percent ratio of the gold nanoparticles at the corresponding locations (1 or [2 + 3 + 4]) shown and marked with the red arrows in cryo‐TEM micrographs (a)–(j) and obtained from the analysis of several images. 1 s and [2 + 3 + 4] s show the simulation results. ***This value represents the maximum number of AuNPs hosted by single polymersome particle detected for each sample.

First of all, AuNPs were encapsulated using the well‐established in situ method. For this purpose, block copolymers with photocross‐linker (details in Figure S2, Supporting Information) were synthesized and assembled using previously reported protocols.^55^ The in situ loading did not significantly affect the size and the dispersity of the polymersomes or the UV–Vis absorbance of the AuNPs (Figures S3a,b and S4 and Table S4, Supporting Information). Cryo‐TEM investigations, depicted in **Figure** **2**, show four distinguishable particle locations at or in the vesicles: adsorbed onto the outer (1) or inner (3) surfaces of the vesicle membrane, trapped directly inside the hydrophobic part of the membrane (2) and loaded into the lumen (4).

Regardless of particle diameter (5 or 10 nm), using the in situ approach more than 80% of the gold nanoparticles were encapsulated in the positions 2, 3, and 4. These locations 2–4 can be considered inside the vesicles. As a next step, the same AuNPs were postloaded in preformed, empty polymersomes. After polymersome formation and cross‐linking, the pH was reduced to 5 and the AuNPs were added. The molar ratio between vesicles and nanoparticles was kept equal to the in situ method with 1:3.8. After 1 h of stirring, the pH was adjusted back to 8 and the vesicles were characterized. Cryo‐TEM images show not only the successful postloading, but they additionally reveal that again AuNPs are observed at locations 2–4 similarly to the in situ loading method. However, in comparison to the in situ method, much more AuNPs are adsorbed onto the outer surface of the vesicles (location 1). This behavior seems reasonable considering the surface charge of each component (Figure S1, Supporting Information). The AuNPs exhibit a negative zeta potential over the entire pH region, while the polymersomes carry positive charge from acidic pH to slightly basic pH due to the protonation of their amino groups. This leads to electrostatic attractions between the AuNPs and the vesicles at pH 5. Hence, zeta potential results of the postloaded AuNP show a clear decrease of the polymersome surface charge in comparison to the in situ loaded polymersomes (Table S4, Supporting Information). This is in agreement with the cryo‐TEM results. An increased modification of the polymersomes surface by the negatively charged AuNPs (location 1) is identified. While 25% of the 5 nm sized AuNPs are located at the outer membrane wall, the larger, 10 nm sized AuNPs lead to significantly higher modification of the outer polymersome surface (≈40%) (Figure 2). Thus, the larger particles are less encapsulated than the smaller AuNPs, although their zeta‐potential is very similar (Table S4, Supporting Information). This observation leads to the conclusion that the size of the cargo is a limiting factor in the case of AuNPs for applying the postloading method. This insight corresponds to data analysis described in the literature^52^, ^53^, ^54^ and expands significantly the variety of known tailoring parameters of loading processes such as the composition and the membrane thickness of the vesicles.

A direct analysis of cryo‐TEM images was performed to determine AuNPs locations based on approaches known from literature.^52^, ^53^, ^54^ However, one should take into account the technical restrictions of TEM that give 2D projections of 3D objects. The visualization of the 3D position of AuNPs would be only possible with a highly elaborate cryo‐tomography. Thus, we considered an interpretation of our data using Monte Carlo (MC) simulation (see the Supporting Information) in order to quantify the relationship between particle counts in locations 1, 2–4 determined from the 2D images (Figure 2) and the particle counts in the same locations in 3D space. The results from the MC simulation show (Figures S5–S9 and Tables S5–S7, Supporting Information) that the number of AuNPs observed in cryo‐TEM images at location 4 is largely overestimated, whereas it is largely underestimated at location 1. This means that a considerable portion of the AuNPs placed in the outer region (location 1) appear in a 2D image inside of the vesicles (apparent location 4). Furthermore, AuNPs at locations 2 and 3 are only slightly deviating from their 3D position according to the MC simulations, thus, their number in 2D projection is reasonable, well matching the number in 3D location.

We should explicitly stress that it is not possible to deduce 3D positions of particular AuNPs in a particular polymersome from a single 2D image of that polymersome, as presented in Figure 2. Instead, the MC simulation provides quantitative relationship (correction factor) between average number of AuNPs at apparent location (1–4) in 2D projection and average number of AuNPs at respective location (1‐4) in 3D projection (Tables S6 and S7, Supporting Information).

The simulation results generally confirm a lower uptake of AuNPs in location 2, 3, and 4 (5.6% for PS‐Au‐10p and 31% for PS‐Au‐5p) after postloading compared to in situ loading (56–61% for PS‐Au5 and PS‐Au10) (Figure 2k). This implies that a higher presence at location 1 for postloaded AuNPs as hard spheres is given, while a further differentiation between locations 2 and 4 is not really possible with the cryo‐TEM study. This size dependency during postloading is presented with different proteins—small myoglobin and large esterase—below.

Nevertheless, one essential result from the combined analysis via cryo‐TEM and MC simulation is that AuNPs are taken up at different locations of the polymersomes depending on the loading procedure. In light of this deduction, the obtained data (Figure 2) give first qualitative and semi‐quantitative values for the locations of AuNPs.

### 2.2 In Situ and Postloading of Hyperbranched Glycopolymers by Polymersomes

Besides the use of well visible AuNPs of high contrast under cryo‐TEM conditions, we aimed at investigating the postloading and in situ loading approach of soft nanoparticles by polymersomes. Therefore, well‐established cationic, spherical dendritic glycopolymers, based on the modification of hyperbranched poly(ethyleneimine) (PEI) with maltose units were used (further details in the Supporting Information). Depending on the size of the dendritic glycopolymers, particles of average diameter of 7 (PEI‐5) and 15 (PEI‐25) nm were obtained. In order to investigate both loading methods and the loading efficiency of dendritic glycopolymers by polymersomes, traceable dendritic glycopolymers PEI‐5 and PEI‐25 (Figure 1) were realized by staining both precursors with rhodamine B. Both dendritic glycoarchitectures are considered protein mimics due to their similarity to natural proteins (e.g., high solubility in physiological environment and noncovalent interactions, e.g., H‐bonds and ionic interaction). The use of protein mimics will help us to understand the natural protein interactions with polymersomes and crossing of polymersomes membrane.

**Figure 3:**
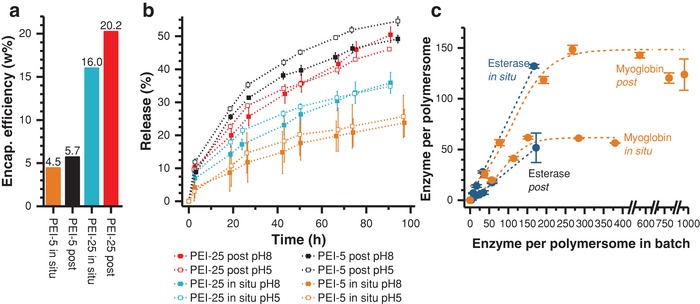
a) Encapsulation (Encap.) efficiency (= weight %) of PEI‐5, PEI‐25 by in situ loading and postloading; determined by a combination of shear force‐driven HFF purification and UV–vis spectroscopy. b) Release kinetics of PEI from polymersomes at pH 5 and 8 given as percentage of encapsulated PEI. c) Loading efficiency of esterase and myoglobin by in situ loading and postloading determined by AF4—using unpurified samples. Further details on loaded enzyme per polymersome as percentage are given in Table S8 (Supporting Information). For example, Myoglobin in situ ranges from ≈15 and ≈41%, when adding different amounts of enzymes to polmersome without purifying the samples for AF4 study. Note that results of Figure 3a,c are not comparable due to differently applied methods with/without purification steps.

We performed in situ loading and postloading procedures at pH 8 and 5, respectively, followed by the purification of dendritic glycopolymer‐loaded polymersomes solution from free PEI‐5 and PEI‐25 by the established hollow fiber filtration (HFF) with defined transmembrane pressure.^24^ Comparing PEI‐5 and PEI‐25 loading efficiency (**Figure** **3**a; Figure S10, Supporting Information), it is clearly visible that PEI‐25 possesses up to 4 times higher loading efficiency as found for PEI‐5.

Regardless of the size of the glycopolymer particles, in situ loading exhibited a lower loading efficiency of about 80% of the postloading approach. It should be also noted, that all possible particle locations 1–4 (Figure 1) can contribute to the calculated amount of loading.

After in situ loading and postloading with dendritic glycopolymers release experiments at pH 5 and 8 for PEI‐5 in situ, PEI‐5 post, PEI‐25 in situ, and PEI‐25 post (Figure 3b; Figures S10–S13, Supporting Information) were performed to gather additional information on the location of particles, especially for the differentiation between the locations 1 and 4 (Figure 1), and on the release kinetics. From the theoretical point of view, we expected different release behavior at pH 5 and 8 for each experimental series (Figure 3b). This difference should result from the collapsed, nonpermeable vesicles membrane at pH 8 and the swollen, permeable vesicles membrane at pH 5. In the swollen membrane state additional amounts of PEI‐5 and PEI‐25 macromolecules should be released by crossing the membrane from the lumen (location 4) outwardly.

However, in all cases we found only small differences in release at pH 5 and pH 8, pointing on a substantial release of both nanoparticles at pH 8 when the membrane is collapsed. However, the release varies essentially with the dendritic glycopolymer size and the loading method (Figure 3b). Higher release is found for the post approach compared to the in situ approach, whereas small differences were found between released PEI‐5 and PEI‐25. Thus, one can conclude that the release is mainly caused by PEI‐5 and PEI‐25 macromolecules located at position 1 (adsorbed at the outer surface of the membrane, see Figure 1) and that the PEI particles located at position 2–4 are not released in a substantial amount at pH 5.

To conclude this part, the uptake of dendritic glycopolymers PEI‐5 and PEI‐25 after postloading leads to a more efficient but less specific loading at the outer surface of polymersomes (location 1) and thus, to higher release as compared to the in situ loading method. Concerning the influence of size on loading characteristics, larger PEI‐25 nanoparticles are loaded to a higher degree (Figure 3a), but less selectively concerning the vesicle's lumen (location 4) compared to the smaller PEI‐5 glycopolymers.

These conclusions are in line with the results on polymersome loading with AuNPs. Surprisingly, both cationic glycoarchitecture particles (Figure S1, Supporting Information) are able to overcome the cationic repulsive force of swollen polymersome membrane in the postloading approach, finally being partially captured in the polymersome lumen (location 4).

### 2.3 Stability of Polymersomes in Presence of Digesting Enzymes

Although a number of different examples for enzymes have been reported in the literature,^56^ the combination of polymersomes and digesting enzymes which are able to break chemical bonds is rather uncommon.^57^, ^58^ In order to progress toward sophisticated systems like “molecular factories” with digesting compartments, the application of bond‐breaking enzymes for polymersomes functions as artificial organelles and cells is essential. Therefore, we explored whether such enzymes are tentatively able to cut ester bonds of block copolymers in polymersomes. If yes, this can subsequently destroy the entire vesicle. Stability tests of empty polymersomes in presence and absence of esterase and proteinase K show (Figure S14, Supporting Information) that polymersomes are stable against both digesting enzymes over several days at low protein concentration (0.02–0.03 mg mL^−1^). Interestingly, for both enzymes higher concentrations (0.2–0.3 mg mL^−1^; also using inactivated enzymes) lead to size increase of the polymersomes. This is a first hint for the adsorption of enzymes on polymersomes surface, also initiating the formation of larger structures (= adsorption of enzymes on polymersome surface or formation of few polymersome dimers; Figure S14, Supporting Information). Moreover, the enzymatic activity of both enzymes does not significantly decrease during the storage time (Figure S15, Supporting Information). With these promising results confirming stable polymersomes in the presence of digesting enzymes, we took a step forward studying the uptake of the proteins myoglobin and esterase by polymersomes as well as the structural parameters of these biohydrid strcutures by AF4.

### 2.4 Loading Efficiency of Enzymes by Polymersomes and Structural Parameters of Polymersomes Investigated by Asymmetrical Flow Field‐Flow Fractionation

The combination of HFF or dialysis as separation methods and absorption spectroscopy for enzyme assays is feasible to prepare and analyze higher sample volumes. Unfortunately, calibrating enzymatic activities and running HFF purifications are tedious and time‐consuming processes under harsh conditions, which do not allow for a deeper interpretation of the cargo interactions with polymeric vesicles, and do not provide information on their size and shape. To overcome these limitations, we applied AF4. Using this method two goals can be achieved: i) separation and quantification of the nonencapsulated cargo from the polymersome and ii) evaluation of the conformation of the polymersome shape in dependence of the loading method, in situ or post.

In contrast to HFF, AF4 (see the Supporting Information for details on the method) is based on much milder and gentler separation approach resulting in minimal destruction of supramolecular assemblies.^59^, ^60^, ^61^, ^62^, ^63^

#### 2.4.1. Study on Empty Polymersomes by AF4

**Figure 4:**
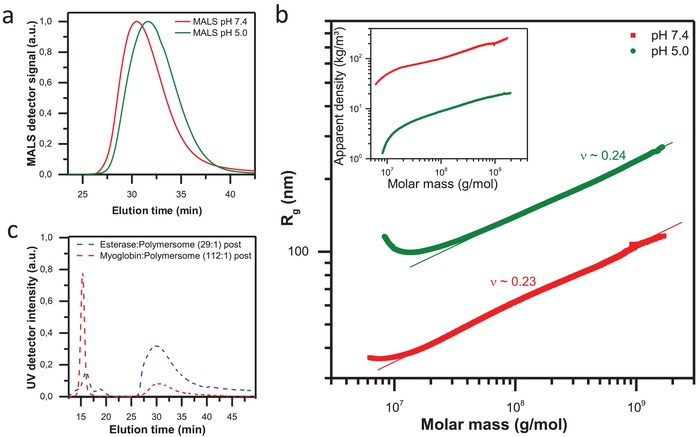
a) Static light scattering signal of polymersome measured at pH 5 (green lines) and pH 7.4 (red lines) after AF4 separation (method A). b) Conformation plot of polymersome measured at pH 5 (green dots) and pH 7.4 (red dots) indicating uniform particle conformation with well‐defined surface. The inset shows the apparent density calculated according to $\rho_{\mathrm{app}}=\frac{M_{w}}{V(R)}$. c) UV signal after AF4 separation of two systems: free esterase and polymersomes postloaded with esterase (blue line); free myoglobin and polymersomes postloaded with myoglobin (red line).

To evaluate changes in the polymersome elution behavior and shape, empty polymersomes as references were precisely characterized before the loading experiments started, using AF4 coupled to static and dynamic light scattering. Different pH values clearly show a shift of the fractogram at low pH to higher elution times (**Figure** **4**a). This corresponds to an increased size of polymersomes due to the swollen state of polymersome membrane. The radius of gyration increases too, while the apparent density decreases, as expected (Figure 4b). The slope of the scaling plot corresponds to the scaling exponent *ν*in the scaling law *R*_g_ = *K*·*M^ν^*, and enables the interpretation of shape and conformation for various particles. For hard sphere in a good solvent *ν*= 0.33 is expected corresponding to 3D fractal object. Though, this value is additionally strongly depending on the nature of particles surface. It was theoretically calculated that values lower than 0.3 would correspond to spherical particles with a smooth, well‐defined surface.^64^ Thus, the conformation plots of the empty polymersomes, determined at pH 5 and pH 7.4, indicate uniform particle conformation with a smooth, well‐defined surface independent on the applied pH.^64^, ^65^, ^66^

#### 2.4.2. Study on Enzyme‐Loaded Polymersomes by AF4

Please note, in contrast to the HFF‐purified samples used in the other experiments, for the AF4 study no purification step was performed to the complexation solution of polymersome and enzyme, thus free enzyme and loaded polymersomes are coexisting in the sample solution. First of all, we developed an adequate protocol to separate two proteins possessing different sizes from the polymersomes. Myoglobin is a small protein with number average molar mass of 16 kg mol^−1^ and 5 nm in diameter, while Esterase is characterized by 185 kg mol^−1^ and 10 nm in diameter. Optimizing the fractionation conditions (method description and Figures S18–S22, Supporting Information), we were able to achieve baseline separation between polymersome and proteins visible in the UV signal (Figure 4c). The calibration of the UV signal area with concentration series of the individual enzymes enables us to quantify free enzyme and to calculate the amount of loaded enzymes by polymersomes (Figures S18–S20 and S40, Supporting Information).

Different polymersome/enzyme ratios for in situ loading and postloading were characterized. Only significant results of the AF4 study are presented here, while the complete AF4 results are collected in the Supporting Information. For comparison of the data between different vesicle and enzyme concentrations, we plotted the number of loaded enzymes per polymersome against the number of enzymes per polymersome in the batch. The molar concentration of the enzymes is easily accessible because their molecular weight was determined via AF4 and multiangle laser light scattering detector (Table S1, Supporting Information). The results of the quantification experiments are depicted inFigure 3c and Table S8 (Supporting Information). First, it should be noted that raising the amount of enzymes leads to an increase in the loading efficiency. Yet, clear differences depending on both, the loading approach and the protein type, were indicated.

The postloading of myoglobin is more effective than the in situ strategy (Figure 3c). In both cases, loading is mainly but not exclusively directed toward location **1,** the outer membrane surface (Figure 1). Obviously, the forces applied during AF4 separation are too weak to break the interaction between the vesicle surface and the cargo biomacromolecules. By contrast, applying high pressures via HFF (Supporting Information), only a small amount of myoglobin biomacromolecules after postloading was indicated. Thus, AF4 experiments confirm the working hypothesis that during postloading, location 1 is stronger occupied than after in situ loading. This was further validated by analyzing the enzymatic activity of the polymersomes (presented below).

Figure 3c additionally shows that after increasing the cargo ratio above 150 myoglobin biomacromolecules per polymersome for in situ loading, no further increase in loading efficiency can be detected. This confirms that all available binding valences per formed polymersomes are occupied.

Loading of esterase leads to the opposite behavior (Figure 3c). For this, larger enzyme in situ loading proves to be more effective than postloading at the same batch ratio. The strong interactions between esterase and the copolymer during the in situ loading lead to extremely high incorporation (≥86%) of esterase into the polymersome. This fact explains the formation of turbid solutions and precipitation at higher amounts of esterase (batch ratio >180 biomacromolecules per polymersome) used for in situ loading. Since no aggregation behavior was observed using AF4, it is obvious that the reason is an immense increase of the molar mass by the confined esterase in the internal space of the polymersome. Such behavior cannot be observed using the postloading (Figure 3c), at which esterase can mainly interact with the membrane surface without passing through it to a significant extend. Deeper analysis of the vesicle's conformation revealed that during in situ encapsulation of esterase, substantial amounts of this enzyme are incorporated into the hydrophobic membrane itself (shown below). This phenomenon is not observed for myoglobin and can be potentially explained by the higher loading efficiency of esterase, using in situ encapsulation (Figure 3c; Table S8, Supporting Information), due to strong membrane/enzyme interactions.

**Figure 5:**
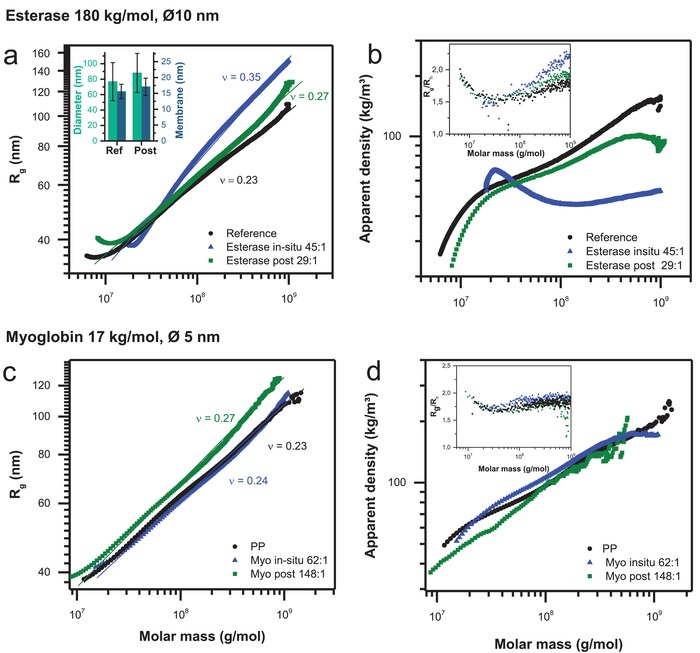
Comparison between pure (black symbols), in situ loaded (blue symbols), and postloaded (green symbols) polymersome. a) Conformation plot of polymersomes: pure, in situ loaded, and postloaded with esterase (esterase in situ and esterase post). Inset: Average values of membrane thickness (blue) and polymersome diameter (green) of an empty polymersome and an esterase post loaded polymersome determined by cryo‐TEM. b) Apparent density calculated for polymersomes: pure, in situ loaded, and post loaded with esterase (Esterase in situ and Esterase post). The inset shows *R*_g_/*R*_h_ dependency on the molar mass: esterase loaded polymersomes show higher ratios after loading especially in situ corresponding to the lower density of the polymersomes after in situ loading and postloading. c) Conformation plot of polymersomes: pure, in situ loaded, and postloaded with myoglobin (Myo in situ and Myo post). d) Apparent density calculated for polymersomes: pure, in situ loaded, and postloaded with myoglobin (Myo in situ and Myo post). The inset shows *R*_g_/*R*_h_ dependence on the molar mass: myoglobin loaded polymersomes do not show any significant change after in situ loading and postloading.

This behavior should have strong impact on the vesicle's conformation. Indeed, the study of the polymersome membrane conformation after enzyme loading shows clear differences depending on the type of protein and loading approach (**Figure** **5**). Most prominent changes of the membrane nature are observed after in situ loading with esterase. The scaling parameter (ν) increases from 0.23 to 0.35 indicating i) that the spherical shape of the particle is remaining after the loading and ii) that the membrane surface changes from smooth and well‐defined in the nonloaded state to membrane with increased roughness in the loaded state (Figure 5a). Obviously, the strong interaction between protein and membrane leads to transformation of the compact, defined copolymer assembly to less homogeneous structure in combination with the protein, thus resulting in increased scaling exponent ν. As a result of the nonregular structure of the membrane, the corresponding apparent density decreases clearly (Figure 5b) confirming this observation.

Postloading of esterase leads to similar changes in conformation and density, yet less pronounced as indicated in Figure 5a,b. Additional, independent validation of the differences in conformation delivers the simultaneous determination of hydrodynamic radius to radius of gyration. The *R*_g_/*R*_h_ ratio is a parameter with values of 0.78 for a hard sphere and increases for well rinsed anisotropic objects. This ratio shows noticeable anisotropy and lower compactness in the membrane conformation after in situ loading of esterase (Figure 5b, inset).

These facts show that the esterase protein is obviously encapsulated to high extent within the membrane during the in situ loading. The large size of esterase biomacromolecules impedes diffusion through the membrane during postloading process and thus enzyme loading is preferably at or in the membrane (location 1 or 2).

cryo‐TEM on postloaded esterase further confirms the trends observed by AF4 (Figure 5a, inset; Figures S41 and S42, Supporting Information). Even by postloading of only 8 esterase biomacromolecules per polymersome cryo‐TEM shows an increase of average vesicle size by 11 nm while simultaneously the membrane thickness slightly increases from 15.6 to 17.1 nm.

The contrary phenomenon is observed when the smaller protein myoglobin is loaded. Myoglobin only insignificantly influences the membrane conformation after loading. In situ loading does not lead to measurable differences in membrane conformation (Figure 5c). By contrast, postloading leads to slightly increased scaling exponents indicating a membrane with more open and rough conformation than the empty polymersome, but significantly less disturbed (even at high loading amounts of 148:1) than in the case of esterase encapsulation. This interpretation is confirmed by both, the scaling and the apparent density behavior of myoglobin loaded polymersomes (Figure 5d). Concluding on myoglobin encapsulation, the influence of size and charge of myoglobin is obviously too small to lead to significant membrane changes, even if the myoglobin biomacromolecules are mainly attached at location 1 of polymersomes after postloading. Obviously, only few myoglobin biomacromolecules diffuse through the membrane after postloading into the lumen (location 4), which was confirmed by enzymatic activity studies shown in the next section. Additionally, this supports the results from the time and pH dependent release experiments of smaller protein mimic PEI‐5 as discussed above (Figure 3b).

### 2.5 Enzymatic Activity of Nanoreactors Fabricated by In Situ Loading and Postloading

**Figure 6:**
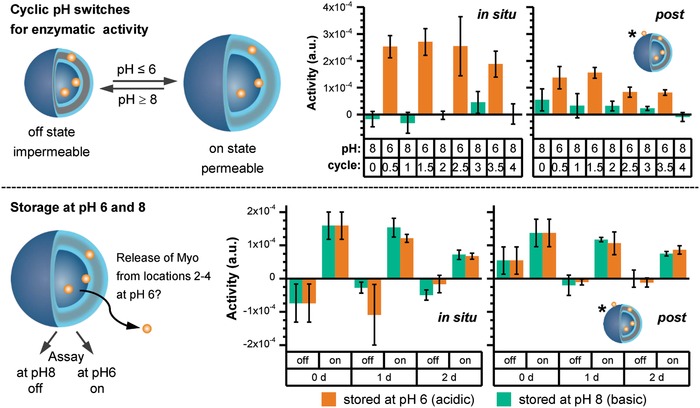
Myoglobin (Myo) encapsulated/loaded in polymersomes comparing in situ loading and postloading samples: cyclic pH switches for enzyme activity (top) and enzyme release during storage at pH 6 and 8 over 2 days (bottom). For storage enzyme assay at pH 6 and 8 (bottom) each sample was stored at pH 6 and 8 over 2 days. For each storage sample at pH 6 and 8, an additional enzyme test was carried out at pH 8 for sample stored at pH 6 and at pH 6 for sample stored at pH 8 at each time point [0, 1, and 2 day(s)]. * = Starting of real experiments with Myo‐polymersomes with location 1 for postloading samples after HFF purification.

As already discussed, polymersomes used in our study are obviously nonsensitive toward digesting enzymes and show different interaction characteritics toward the proteins myoglobin and esterase. Now, the question appears, to which extent are these enzymes active depending on the loading procedure and can we draw additional conclusions about their location from their activity profile. Previous studies by Gräfe et al. demonstrated impressively that in situ loading of enzymes into our polymersomes (Figure 1) is useful to construct nanoreactors with “on and off”‐switchable enzymatic cascade reactions.^67^ Here, we validate and expand the previous in situ loading results with the postloading approach in respect to i) cyclic pH switches for enzymatic activity and ii) enzymatic storage activity at different pH values (**Figure** **6**). This includes the further understanding on the enzyme cargo location and the indication on whether enzymes can escape from the polymersome lumen during storage in acidic environment (Figure 6).

For the final experiment series, myoglobin was chosen as commonly used, small enzyme with 5 nm diameter and was encapsulated in the polymersomes using the in situ loading and the postloading approach. For both loading approaches the same amount of myoglobin was used. Successful encapsulation by the polymersomes was proven by cyclic enzyme activity switching experiments between pH 6 and 8 (Figure 6, cyclic pH switches). Free myoglobin in the solution was removed by a HFF purification step before starting any enzymatic experiments. For these studies, the fact was used that switching the polymersome membrane from impermeable at pH 8 to permeable at pH 6 (Figure 6), small molecules like hydrogen peroxide and guaiacol can cross the membrane, which allows myoglobin activity detection (Figures S43 and S44, Supporting Information). At pH 8, the membrane is in a collapsed, shrunken state and therefore not permeable for at least one of the mentioned substrates. The results in Figure 6 (cyclic pH switch) show that it is possible to switch the enzymatic activity “off” at pH 8 and “on” at pH 6 four times after the use of both in situ loading and postloading samples. The experiment also indicates thatthe postloaded sample is less active at pH 6 compared to the in situ loaded sample due to the lower myoglobin uptake in polymersome lumen triggered by the hurdle of membrane crossing from outside to inside during the postloading procedure. Thus, as already found for postloaded dendritic glycopolymers, we indicate a similar behavior for the cationic myoglobin biomacromolecules at low pH (Figure S1, Supporting Information): A preferential location of the protein at location 1 in postloading is postulated. To validate enzymatic activity of our nanoreactor, a HFF purification step is needed. We indicate a reduced activity compared to in situ loading, but still confirming a valid enzyme loading. Thus, both loading methods are suited to take up myoglobin inside of the polymersomes at location 4 for myoglobin (Figure 1). It is noteworthy that postloading method is accompanied by an adsorption of myoglobin at the outer surface of the polymersomes (location 1), which cannot be completely removed by the HFF purification step. Please also note that without HFF purification, this enzyme was well detected using AF4 (Figure 3c). Therefore, in the beginning low enzymatic activity is observed at pH 8, at which polymersome membrane is collapsed, which disappears over the cyclic pH switches (Figure 6, cyclic pH switch).

After successful encapsulation of myoglobin in photocross‐linked polymersomes using both loading methods, we were curious about the release process of myoglobin itself through swollen polymersomes at pH 6 over a defined swelling period. For this purpose, we constructed a release experiment for comparing the storage at pH 8 (collapsed membrane) and 6 (open membrane) (Figure 6, storage). Using the same prepared and purified samples as applied for cyclic pH switches (Figure 6, cyclic pH switch), vesicles in situ loaded and postloaded with myoglobin were stored for 2 days at basic conditions or at acidic conditions (Figure 6, storage, and Figures S43–S45, Supporting Information). The enzymatic activity was measured daily for each sample at pH 6 and 8, respectively.

One preconsideration of storage experiment was that if myoglobin would escape from the swollen polymersome into the environment during the storage of 2 days at pH 6, the overall enzymatic activity measured at pH 8 should increase. We observed the same enzymatic behavior as found for cyclic pH switches and no increase in the enzymatic activity at pH 8 (Figure 6, storage). Due to use of hydrogen peroxide for the enzyme assay, there is a slight formation of bubbles inside the cuvettes at basic conditions. This leads to an increased light scattering of the samples. This in turn results in noisy baselines and in some negative enzymatic activities and larger standard deviation at basic conditions. Finally, these results undoubtedly indicate that myoglobin passes the membrane during postloading at pH 5 but remains inside at pH 6 over longer time. Again, nonencapsulated myoglobin is smoothly removable in case of in situ loading method, but not in case of postloading method. This is reflected in the low enzymatic activity at pH 8 at the starting point (0 days). The most surprising result of this storage experiment is that in both loading processes encapsulated myoglobin is not released. This can be explained with the fact that at pH 5 the diameter of swollen polymersome is larger than at pH 6.^24^, ^42^ This may include a lower permeability of swollen polymersome at pH 6. One other possible explanation is that the osmotic pressure difference between inside and outside of the polymersomes is not high enough to initiate the next transmembrane transport from inside to outside. In this context, enzymes are thermodynamically trapped inside the vesicles and favor their local confined environment also supported by other existing forces (repulsive forces and ionic interactions) with various impacts on the polymersome membrane and myoglobin. Similar observations have been reported for the entrapment of enzymes in metal–organic frameworks.^68^, ^69^, ^70^ The storage studies at pH 6 and 8 further support the proposed enzyme locations 4 and 1, but also they confirm the postloading results of the protein mimics PEI‐5 and PEI‐25 and their release profiles. Finally, the effect of enzyme trapping at pH 6 for both loading methods can lead to exciting new approaches in mimicking complex cell functions as well as in vesicle‐based bionanotechnology.

### 2.6 Comparing the Results and Concluding Remarks on Loading Processes

This study provides deeper insights into the in situ loading and postloading of photocross‐linked polymersomes with solid and soft nanoparticles. Most studies on in situ loading of proteins by polymersomes mainly consider the final validation of loading efficiency,^71^, ^72^ but not the verification of nanoparticle locations within the molecular structure of the polymersome itself and the consideration of the structural parameters of polymersome in presence and absence of loaded nanoparticles and proteins, except for transmembrane proteins.^33^, ^73^ Moreover, postloading of nanoparticles into polymersomes has been rarely studied in detail, even though the potential use of protein postloading into polymeric vesicles and their multicompartments may open new perspective in different application fields. This is highly needed, e.g., to establish more complex and sophisticated cell mimics for example for enzymatically triggered therapeutics.

After studying in depth the loading characteristics of our photocross‐linked polymersomes, the following can be stated:

Generally, the postloading approach leads to a successful loading of all kinds of nanoparticles (Au, dendritic glycopolymers, and enzymes with nanometer‐sized dimension between 5 and 15 nm) (Figure 1). Thus, it emphasizes an alternative to the in situ loading process allowing the uptake of additional nanoparticles by the fully swollen polymersomes within the membrane (location 2), inside the lumen (locations 3 and 4) and outside of the membrane (location 1).

The post/in situ loading and release study of dendritic glycopolymers as protein mimics shows that in situ loaded nanoparticles exhibit a lower release characteristics compared to postloaded nanoparticles. This implies a higher presence of postloaded nanoparticles at locations 1 and 2 compared to locations 3 and 4, while for in situ loaded nanoparticles the opposite situation is more favored. The preferred locations triggered by post/in situ loading process are also confirmed for the loading with real enzymes. Reversibly pH switchable enzymatic nanoreactor, at which the enzyme is in situ loaded, shows slightly superior enzyme activity compared to that with postloaded enzymes (Figure 6, top). The studies demonstrate that we can distinguish clearly between the locations 1 (outer surface of polymersome membrane) and 4 (polymersome lumen), but not between locations 1 and 2 or 3 and 4.

**Figure 7:**
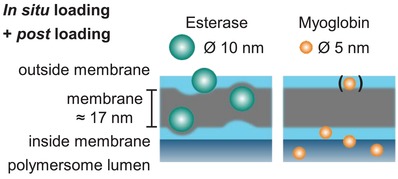
Schematic representation of protein location and membrane deformation/integration as indicated by AF4 measurements (Esterase: higher scaling parameter ν and lower apparent density to empty polymersomes; Myoglobin: no significant differences compared to empty polymersomes) and myoglobin release studies.

Further insight into the membrane integration (location 2) of myoglobin in enzymatic nanoreactors has been achieved by AF4, when the scaling parameter ν is considered for empty, in situ loaded and postloaded polymersomes. The membrane integrity and apparent density of polymersomes are not essentially influenced for in situ loaded polymersomes. This indicates that obviously there is no or a very low amount of the small myoglobin integrated in the membrane of the enzymatic nanoreactors (**Figure** **7**). A similar situation is also observed for postloaded myoglobin in polymersomes. The slightly higher ν value is related to a stronger interaction of myoglobin with the outer membrane of polymersomes during the postloading process, but membrane integration of myoglobin at location 2 is possibly taking place only to a low degree. No significant change in the membrane integrity can be observed when considering the apparent density of postloaded polymersomes. The obvious absence of myoglobin at location 2 is supported by the release and stability experiments of swollen myoglobin containing nanoreactors at pH 6, since no myoglobin is released from the nanoreactor after the first cycle.

In contrast to myoglobin's location after in situ loading and postloading, results from AF4 undoubtedly proof strong membrane integration (location 2) of esterase due to the presence of larger scaling parameter ν and decreasing apparent density for in situ loading and postloading compared to not loaded polymersomes. The conformational characteristics of myoglobin for postloading confirm similar behavior at lower extent. Cryo‐TEM study also reveals the membrane integration of esterase after postloading approach, even at very low postloading ratio of 8 protein equivalents toward one polymersome. Thus, the AF4 study smoothly exemplifies that a differentiation between membrane‐integrating protein esterase and nonmembrane‐integrating protein myoglobin is possible. In situ loaded and postloaded esterase is undoubtedly present in location 2 while myoglobin did not integrate in the polymersome membrane to a significant amount by both loading processes (Figure 7). Yet, postloading of myoglobin into a swollen polymersome mainly leads to stable, entrapped cargo into the lumen of the polymersome.

Generally, it can be concluded that the postloading process of both enzymes promotes the desired transmembrane diffusion into the polymersome lumen at pH 5 in presence of cationic repulsive charge for both enzymes and polymersome membrane, but also all other soft and solid nanoparticles can cross the cationic polymersome membrane independent on size and charge. Lower amounts of larger nanoparticles, especially Au NP with *Ø* of 10 nm and PEI‐25 with *Ø* of 14.7 nm, can successfully cross the polymersome membrane reaching the lumen during the postloading process. Smaller soft and solid nanoparticles (*∅* ≤ 7 nm) can be postloaded in the polymersome lumen at slightly lower degree as found for in situ loaded nanoparticles. This means that size and charge of these nanoparticles (Au NP, PEI‐5, and myoglobin) are playing a secondary role for crossing the swollen, cationic polymersome membrane, whereas the softness of nanparticles plays a dominate role in the postloading process. It also defines whether the particles cross the membrane or are integrated into the membrane, when comparing the membrane integration of esterase (*∅* 9.8 nm) and AuNP (*∅* 10 nm) during the postloading process. It is obvious that the soft protein esterase with its highly flexible polypeptide scaffold can be smoothly integrated in polymersome membrane, while the solid AuNPs are only able to cross the swollen polymersome membrane to a low degree and are negligibly integrated into the membrane.

## 3 Conclusion

Protein trapping and surface decoration after postloading of swollen polymersomes can pave the way of exciting new approaches in polymeric vesicle‐based bionanotechnology. In this development, the combination of advanced multidetection separation approaches contributes significantly to the understanding of these processes. Although the postloading selectivity toward the lumen encapsulation is slightly limited due to the nanoparticles softness and size in comparison to the in situ loading, the post approach enables tuning the characteristics of loaded polymersome very precisely, and thus the controlled loading of polymersomes is unlocked. This emphasizes a new and valuable tool in the manifold applications of multiresponsive, swellable polymersomes in the field of protocells.

## 4 Experimental Section

*Materials*: The block copolymer (BCP1 composition presented in Figure S2, Supporting Information) used for polymersome fabrication to carry out in situ loading and postloading process of dendritic glycopolymers (PEI‐5 and PEI‐25) and enzymes was synthesized like previously reported (further details of characterization in the Supporting Information).^24^, ^42^ The block copolymer BCP2 having other cross‐linking units (Figure S2, Supporting Information) for encapsulation of gold nanoparticles was synthesized as reported elsewhere.^55^ Porcine liver esterase, myoglobin (from equine skeletal muscle) and guaiacol were purchased from Sigma‐Aldrich and hydrogen peroxide (30%) was purchased from VWR. Gold nanoparticles (5 and 10 nm) were purchased as suspension in 0.1 × 10^−3^ m phosphate buffered saline (PBS) solution from Sigma‐Aldrich. All substances were used as received.

*AF4*: AF4 measurements were performed with an Eclipse DUALTEC system (Wyatt Technology Europe, Germany) with 0.01 m PBS buffer as carrier liquid and 0.02% (w/v) NaN_3_ to prevent growth of bacteria and algae. The channel spacer made of poly(tetrafluoroethylene) had a thickness of 490 µm, and the channel dimensions were 26.5 cm in length and from 2.1 to 0.6 cm in width. The membranes used as accumulation wall were composed of regenerated cellulose with a molecular weight cut off (MWCO) of 10 kDa (Superon GmbH, Germany). Flow rates were controlled with an Agilent Technologies 1260er series isocratic pump equipped with vacuum degasser. The detection system consists of a MALS detector (DAWN HELEOS II, Wyatt Technology Europe, Germany) operating at a wavelength of 690 nm with online DLS detector (DynaProNanoStar, Wyatt Technologies, USA) which is an add‐on unit connected to the 99° angle of the MALS, a variable wavelength detector (1260 series, Agilent Technologies Deutschland), a variable wavelength detector (VWD, 1260er series, Agilent Technologies, Germany), and a refractive index (RI) detector (Optilab T‐rEX, Wyatt Technology Europe GmbH, Germany) operating at a wavelength of 658 nm. All injections were performed with an autosampler (1260 series, Agilent Technologies Deutschland GmbH). The data collection and calculation of molar masses and radii were performed by Astra 6.1.2.84 software (Wyatt Technologies, USA).

The channel flow rate (*F*_c_) was maintained at 1 mL min^−1^ for all AF4 measurements. If not mentioned otherwise, the focus flow (*F*_f_) was set at 1.5 mL min^−1^ for 8 min. The injection volume for molar mass determination was set to 100 µL and for online DLS it was increased to 200 µL.

Depending on the studied system, different measurement profiles were applied. Polymersome samples with esterase were separated by following parameters (separation strategy A): the separation starts with an isocratic step with a cross flow rate (*F*_x_) of 2 mL min^−1^ for 15 min followed by a linear *F*_x_ gradient from 2 to 0 mL min^−1^ within 2 min. The last step proceeds without *F*_x_ (0 mL min^−1^) for 25 min. In case of esterase, the VWD was set to 275 nm.

For the separation of samples with polymersomes and myoglobin (separation strategy B), only the starting *F*_x_ rate was increased to 5 mL min^−1^ and the VWD was set to 410 nm. All other parameters stayed the same.

For the calculation of enzyme amounts, three different concentration series of pure enzyme solutions were injected (100 µL of varied concentration) and separated by the specific separation profile (strategy A for esterase and B for myoglobin). Recovery tests with varied sample loads verified that no adsorption takes place at fresh membranes.

*Loading of Gold Nanoparticles by Polymersomes*—*In Situ Loading of Gold Nanoparticles*: Block copolymer BCP2 was dissolved in aqueous HCl solution (pH 2) then passed through 0.2 µm nylon filter. Afterward, pH was increased to a value of pH 5 by utilizing 1 m NaOH solution. The addition of gold nanoparticles (in 0.01 × 10^−3^ m phosphate buffer, 5 or 10 nm) was performed dropwise along with constant stirring. Afterward, the pH was increased to pH 8 to obtain the polymersome/gold nanoparticle assemblies followed by stirring four days in dark condition. Finally, the photocross‐linking of polymersome membrane was carried out for 30 min using the UV chamber equipped with an iron lamp (UVACUBE 100, honle UV Technologies, Germany). The total block copolymer concentration was kept as 1 mg mL^−1^ and the molar ratio of gold NPs to block copolymers was adjusted as 3.8. For purification of nonencapsulated gold nanoparticles; hollow fiber filtration with modified polyethersulfone based filter modules (MWCO: 750 kDa, SpectrumLabs, USA) was used. The transmembrane pressure was maintained as 150 mbar during the filtration and phosphate buffer at pH 8 was used as an eluent for several cycles of filtration process. Postloading of gold nanoparticles—herein, first the polymersomes (1 mg mL^−1^) were prepared as described previously. Afterward, the pH of the polymersomes was gradually decreased to pH 5 by addition of HCL (0.1 × 10^−3^ m) solution, which led to the swollen state of the polymersomes. By following this, the addition of gold nanoparticles (in 0.01 × 10^−3^ m phosphate buffer, 5 or 10 nm) to the acidic polymersomes was carried out dropwise along with constant stirring. In order to provide sufficient time for gold nanoparticles to diffuse into the polymersome lumen, the polymersome/gold NPs mixture was stirred for an hour before pH was increased to the basic state (pH 8). The adjustment of the pH was performed by slow addition of NaOH solution (1 m). Herein, the molar ratio of gold NPs to block copolymers was kept as 3.8. The final block copolymer concentration after gold nanoparticle incorporation was 0.64 and 0.66 mg mL^−1^ for PS‐Au10P and PS‐Au5P, respectively.

*Loading and Release of Dendritic Glycopolymers by/from Polymersomes*: In situ loading of dendritic glycopolymers PEI‐5, an PEI‐25 by polymersomes—1 mg of each PEI macromolecule per mL of BCP1 solution was added before raising the pH to 8–9 for starting the assembly procedure. The mixture was stirred in the dark for three to four days, passed through a syringe filter (cellulose ester, 0.8 µm), and cross‐linked in small aliquots for 90 s each. Afterward, HFF was performed to separate nonencapsulated PEI. For postloading of PEI, macromolecules polymersomes were prepared and cross‐linked according to the standard method. Afterward, 1 mg PEI macromolecules per 1 mL of polymeric vesicle solution were added before lowering the pH down to 5 by addition of HCl followed by overnight stirring. Then, the pH was brought back to 8 by addition of NaOH. Afterward, HFF is performed.

For HFF separation, 7.5 mL of the PEI‐containing polymersome solution was transferred into a falcon tube and diluted up to a total volume of 50 mL with PBS buffer at pH 8 (PBS‐8). A membrane module with a MWCO of 300 kDa was used. HFF was executed while the sample volume was kept constant at 50 mL by the addition of fresh PBS‐8. When 100 mL of PBS‐8 was added, the sample volume was concentrated down to 40 mL, where it was kept constant by PBS‐8 addition until 60 mL of PBS was added. Then, the sample was concentrated down to 30 mL total volume, which was kept constant by the slow addition of 40 mL PBS‐8. Afterward, the sample volume was decreased to 20 mL which was kept constant for another 20 mL of PBS‐8. Finally, the sample was concentrated down to 15 mL total volume in the end. The transmembrane pressure was kept at a maximum of 120 mbar during the whole process.

For release study aliquots of 2 mL of the PEI‐containing vesicles were transferred to the dialysis cap of a “Slide‐A‐lyzer” (ThermoScientific), which was equipped with a dialysis membrane of a 20 kDa weight cut‐off. The buffer reservoir of the Slide‐A‐lyzer was filled with PBS buffer of the desired pH and was exchanged daily. Twice a day the vesicle solution was removed from the cap and analyzed by UV/Vis spectroscopy (370–800 nm). The solution was then transferred back into the dialysis cap. This procedure was continued for 5 days.

*Myoglobin Loading by Polymersomes*: For in situ loading of myoglobin, a method of Gräfe et al. was adopted and modified.^67^ Myoglobin was solved in PBS buffer at 1 mg mL^−1^. The BCP1 was dissolved at 1.25 mg mL^−1^ in 10 × 10^−3^ m hydrochloric acid (pH 2). The pH of the BCP1 solution was raised slightly to ≈3 followed by the addition of the myoglobin solution (20 vol%), so that finally a polymer concentration of 1 mg mL^−1^ and a myoglobin concentration of 0.2 mg mL^−1^ were achieved. The solution was than assembled and cross‐linked according to the standard protocol. HFF was performed for separation purposes. For postloading of myoglobin, 3.4 mg mL^−1^ of myoglobin was dissolved in PBS. An appropriate amount of this solution was added to the assembled and cross‐linked polymersomes in this manner that finally a myoglobin concentration of 0.2 mg mL^−1^ was reached, whereas the polymer was only slightly diluted to ≈0.94 mg mL^−1^. Afterward, the pH was lowered down to 5 by addition of HCl followed by overnight stirring. Then, the pH was brought back to 8 by addition of NaOH. Afterward, HFF was performed.

For HFF separation, 10–15 mL of the myoglobine‐containing polymersome solution were transferred into a falcon tube and diluted up to a total volume of 50 mL with PBS buffer at pH 8 (PBS‐8). A membrane module with a MWCO of 500 kDa was used. HFF was executed until the sample volume was decreased to 30 mL followed by addition of 20 mL fresh PBS‐8. HFF was continued until a sample volume of 20 mL was reached followed by adding another 20 mL of PBS‐8. HFF was further continued until reaching the original sample starting volume. Now another 20 mL of fresh PBS was added. The last cycle was executed twice, leading to the total sum of ≈120 mL extracted volume. The transmembrane pressure was kept close to, but slightly below, 180 mbar during the whole process.

*Myoglobin Enzyme Activity*: The assay for determining enzymatic activity of myoglobin was adopted from Gräfe et al. with very slight modifications.^67^ In short, 300 µL of sample solution was transferred into a microcuvette and 8 µL of hydrogen peroxide solution (1 m in millipore water) and 8 µL of guaiacol solution (0.1 m in PBS) were added. A lid was placed on top, then the mixture was vigorously shaken and placed in the UV/Vis spectrometer. The absorbance at 470 nm was measured every 12 s for a duration of 3–6.5 min, regarding the amount of enzymatic activity in the solution. Each assay was executed in triplicates.

*Myoglobin and Esterase Loading for AF4 Study*: For in situ loading of myoglobin or esterase, the BCP1 was dissolved at 1.2 to 1.5 mg mL^−1^ (depending on the amount of enzyme stock solution added later) in 10 × 10^−3^ m hydrochloric acid. These solutions were mixed with enzyme stock solutions (3.1 mg mL^−1^ esterase; 0.5–1.0 mg mL^−1^ myoglobin) and millipore water to yield the desired enzyme concentration as well as a BCP1 concentration of 1 mg mL^−1^. Afterward, the pH was changed to 8–9 and the solutions were stirred in the dark for 3 days. Afterward, the vesicles were cross‐linked for 90 s and investigated by AF4. For postloading of esterase or myoglobin, different amounts of vesicle solution (at pH 5), enzyme stock solution (0.5–5.0 mg mL^−1^ esterase; 0.5–1.0 mg mL^−1^ myoglobin, both in 10 vol% PBS‐5), and 0.6 mL of 5× concentrated PBS‐5 were mixed and diluted up to 3 mL overall volume with millipore water (at pH 5). Please note that PBS‐5 refers to PBS buffer, whose pH value was adjusted to pH 5 by the addition of HCl.

A typical example contains 1.5 mL vesicle solution (of 1 mg polymer per mL), 0.3 mL myoglobin solution (0.5 mg mL^−1^ myoglobin), 0.6 mL 5× PBS, and 0.6 mL of water (pH 5), but stock concentrations of enzymes were varied to reach appropriate sample volumes. After mixing, all samples were set to pH 5 and stirred overnight. Afterward, pH values were adjusted to 7.4 and samples were investigated by AF4.

## Conflict of Interest

The authors declare no conflict of interest.

## Supporting information

Supplementary
