# Supplementary material for: Toward Functional Synthetic Cells: In‐Depth Study of Nanoparticle and Enzyme Diffusion through a Cross‐Linked Polymersome Membrane
Source: Adv Sci (Weinh). 2019 Jan 11;6(7):1801299. doi: 10.1002/advs.201801299 (PMC6446602; doi:10.1002/advs.201801299)
Supplement: Supplementary file 1 — Supplementary [file ADVS-6-1801299-s001.pdf]

Copyright WILEY-VCH Verlag GmbH & Co. KGaA, 69469 Weinheim, Germany, 2019.

ADVANCED SCIENCE
Open Access

Supporting Information

for *Adv. Sci.,* DOI: 10.1002/advs.201801299

Toward Functional Synthetic Cells: In-Depth Study of Nanoparticle and Enzyme Diffusion through a Cross-Linked Polymersome Membrane

Hannes Gumz, Susanne Boye, Banu Iyisan, Vera Krönert, Petr Formanek, Brigitte Voit, Albena Lederer, and Dietmar Appelhans**

Copyright WILEY-VCH Verlag GmbH & Co. KGaA, 69469 Weinheim, Germany, 2019.

Supporting Information

Towards functional synthetic cells: In-depth study of nanoparticle and enzyme diffusion through a crosslinked polymersome membrane

Hannes Gumz, Susanne Boye, Banu Iyisan, Vera Krönert, Petr Formanek, Brigitte Voit, Albena Lederer* and Dietmar Appelhans*

Leibniz-Institut für Polymerforschung Dresden e.V., Hohe Straße 6, 01069 Dresden, Germany

E-mail: applhans@ipfdd.de; lederer@ipfdd.de

Dr. H. Gumz, Prof. B. Voit, Dr. A. Lederer

Technische Universität Dresden, School of Science, Faculty of Chemistry and Food Chemistry, 01069 Dresden, Germany

Dr. H. Gumz, Prof. B. Voit

Cluster of Excellence "Center for Advancing Electronics Dresden", Technische Universität Dresden, 01062 Dresden

Dr. B. Iyisan

Max-Planck-Institute for Polymer Research, Ackermannweg 10, 55122 Mainz, Germany

Contents

1 Experimental 3

1.1 Materials and methods 3

1.2 Fluorescence modification of PEI-5 and PEI-25 3

1.3 Polymersome formation and crosslinking 3

1.4 Evaluation of projection effect in electron microscopy by Monte Carlo simulation....4

2. Background Information........6

2.1 Composition and function of photo-crosslinked polymersomes........6

2.2 Principles of AF4 6

2.3 Principles of Static light scattering 7

2.4 Principles of Dynamic light scattering 8

3 Additional Figures and Tables........ 9

3.1 Size, molar mass and zeta-potential of the used cargo nanoparticles and biomacromolecules 9

3.2 Parameters of block copolymers used for polymersome formation........10

3.3 AuNP encapsulation........ 11

3.4 Monte Carlo simulation for the determination of AuNPs in........13 three-dimensional confinement of polymersomes

3.5 Uptake and release of PEI glycopolymers 18

3.6 Polymersome stability against enzymes........ 20

3.7 AF4 investigations 22

3.7.1 Empty polymersomes........ 22

3.7.2 Separation of polymersome and enzymes........ 23

3.7.3 Polymersome loaded with Esterase - in situ encapsulation........ 25

3.7.4 Polymersome loaded with Esterase - post encapsulation........ 27

3.7.5 Polymersome loaded with Myoglobin – in situ encapsulation........ 29

3.7.6 Polymersome loaded with Myoglobin - post encapsulation 31

3.7.7 Quantification of loading capacity by AF4........ 33

3.8 Cryo-TEM........ 35

3.9 Uptake and release of Myoglobin by/from polymersome........36

4 Supplementary References........ 39

1 Experimental

1.1 Materials and methods

Most of the devices and methods have been previously reported.[1-3] Crosslinking of the vesicles has been carried out using a custom made UV chamber connected to an OmniCure S2000 (Excelitas Technologies) device equipped with a high pressure mercury lamp at an irradiation level of 40 W/cm^2. Regarding the second set of polymersomes and AuNP encapsulation the photo cross-linking was carried out for 30 minutes using the UV chamber equipped with an iron lamp (UVACUBE 100, Honle UV Technologies, Germany). For this device the intensity of the light was measured with a power meter (Coherent Fieldmax II TO, USA) and found as 80 mW/cm^2. The photo-crosslinking was performed in round shaped-glass vials in which the path length of light is adjusted as 1.5 cm.

Hollow Fiber Filtration (HFF) is executed utilizing a KrosFlo-Research-IIi apparatus (SpectrumLabs) featuring HFF modules made of modified polyethersulfone while setting the flow rate to 15 ml/min. Zeta-potential titrations and dynamic light scattering measurements were conducted using a Zetasizer nano-Series (Malvern Instruments) device equipped with an automatic titrator using 0.1 M hydrochloric acid.

CryoTEM images were recorded on a Libra 120 (Carl Zeiss). Polymersome samples were adjusted at pH8 and were absorbed on Lacey-type gold grids, frozen in liquid ethane using a grid plunger (Leica Microsystems) and transferred into the microscope using a Gatan 626 cryo holder. UV-vis measurements were carried out using a Specord 210 Plus UV-vis spectrophotometer (Analytik Jena, Germany). Disposable UV cuvettes (Brand, Germany) in micro and half-micro size range were used.

1.2 Fluorescence modification of PEI-5 and PEI-25

100 mg of the maltose modified PEI-precursor (PEI-5-Mal-B and PEI-Mal-B 25 synthesized and characterized according to supplementary reference 4) are dissolved in 5 ml of water, while 17.8 mg Rhodamine B isothiocyanate are solved in 1 ml of DMSO. The solutions are combined and stirred overnight in the dark. Afterwards the reaction is mixed is purified using a short Sephadex column (G25, fine) using water as eluent. After freeze drying 117 mg of the product are isolated.

For the modification of PEI-25 (synthesized according to reference 4) only 7.5 mg of Rhodamin~B isothiocyanate are used per 100 mg of dendritic glycopolymer. For the modification the same procedure is applied as for PEI-5. 105 mg of the product are finally isolated.

1.3 Polymersome formation and crosslinking

The assembly and crosslinking procedure has also been previously reported.[1–3] In general, the block copolymer is solved in diluted hydrochloric acid at pH 2 at a concentration of 1 mg/ml. The solution is passed through a syringe filter (Nylon, 0.2 µm) and titrated with sodium hydroxide solution until pH 8-9 is reached. The mixture is stirred in the dark for three to four

days, passed through a syringe filter (cellulose ester, 0.8 μm) and crosslinked in small aliquots for 90 s each.

1.4 Evaluation of projection effect in electron microscopy by Monte Carlo simulation

The polymersomes loaded with AuNPs are three-dimensional (3D) objects, which are projected in an electron microscope into a two-dimensional (2D) plane – a 2D image. The projection leads to an apparent (falsified) location of AuNPs in polymersome in 2D cryo-TEM images, which is different form the location in three dimensions. In this section we address this problem by means of Monte Carlo (MC) simulations.

To visualize the projection effect more clearly, we start with projection of 2D polymersome with seven AuNPs into a 1D image as depicted in Figure S5. Particle b located in the outer shell of the 2D polymersome appears to be located in the inner shell (*b'*) in the 1D projection. Particles d,e,f located in the inner shell, outer shell and membrane (respectively) of the 2D polymersome, all appear to be located in the lumen (d', e', f') in the 1D projection. Particle a located in membrane of 2D polymersome is projected into correct location (*a'*, membrane) of the 1D projection. Particles c, g are projected in their respective correct locations as well (*c'*, *g'*). The locations of the AuNPs as depicted in Figure S5 are summarized in Table S5.

By adding another 9 simulated polymersomes, each containing 7 AuNPs, the table Table S5 is extended by 9 lines, giving Table S6. The average number of the particles in each part of the 2D polymersome and in each part of the 1D projection is written in the last but one line of Table S5. The last line of Table S6 contains the corresponding standard deviations.

The means and standard deviations can be plot in a graph with average number of particles in 2D polymersome on x axis and average number of particles in 1D projections on y axis. (Figure S6a). Red and blue points lie close to a line with slope 1 ("1:1 line"), which means that the average number of AuNPs located in the membrane and in the inner shell (locations *2'*, *3'*) in the 1D projections are close to the average number of AuNPs located in membrane and inner shell, respectively, in the 2D polymersome (locations 2, 3). Black point lies well below the 1:1 line, which means that the number of AuNPs located in the 1D projection of the outer shell (location *1'*) is considerably smaller than the number of AuNPs located in the outer shell of the 2D polymersome (location 1). In other words, in 1D image we see considerably less AuNPs than they are really present in the outer shell of the 2D polymersome. Since the green point lies well above the 1:1 line, we see in the lumen of the 1D image (location *4'*) considerably more AuNPs than they are really present in the lumen of the 2D polymersome (location 4).

Such simulation can be repeated for 1, 2, 3, … 10 AuNPs in hundreds of polymersomes and can be plotted in one graph.

The above described procedure considered 2D polymersomes and 1D projections for a simplified explanation of the following steps. To investigate the projection effect quantitatively in 3D, we proceed analogously to the 2D/1D case described above, but with 3D

polymersomes and 2D projections (images). We generate hundred PS with random distributions of AuNPs, project them into a 2D image, count the number of AuNPs in the apparent locations in the 2D image and compare them to the counts of the particle locations in 3D. Figure S7 shows the results of 10 simulations with 100 polymersomes for the following parameters of the simulation:

Number of AuNPs per PS:	n = 1, 2, 3, … 9, 10
Inner radius of the membrane:	R_1 = 40 nm
Outer radius of the membrane:	R_2 = 60 nm
Thickness of the inner and outer shell:	t = 10 nm

The slopes of the lines fit in data in Figure S7 give the correction factors. The slope of black line in Figure S7b is 0.38. The number of AuNPs in location 1 in 2D images is on average 0.38 times smaller than in 3D. Thus, the fraction of AuNPs in location 1 in 3D is the fraction of AuNPs in 2D images in location 1 divided by 0.38. Analogously, the fraction of AuNPs in locations 2+3+4 in 3D is the respective fraction in the 2D images divided by 1.45 (slope of the purple line).

The data in Figure S7 are our benchmark model, which we used to test the sensitivity of simulations to various parameters. For results presented in Table S7 we used parameters R_1 and R_2 determined as average sizes from cryo-TEM images (Figure 2 in paper): for specimens PS-Au5, PS-Au10, PS-Au5p, PS-Au10p: R_1 = 84 nm, 92 nm, 92 nm, 98 nm, respectively. R_2 = R_1 – 17 nm (same membrane thickness for all polymersomes). The thickness t of the inner shell and outer shell was considered as unknown parameter having values of 5 nm, 7.5 nm or 10 nm.

In the first step we assumed uniform distribution of the AuNPs, i.e. no preference of AuNPs for location 1 or 2 or 3 or 4. Using corresponding MC simulations we calculated correction factors for all 4 specimens for parameters t = {5, 7.5, 10} nm (Figure S8). We used the correction factors from Figure S8 and fractions of AuNPs in locations 1 and 2-4 in cryo-TEM images (Figure 2; paper) to calculate fractions of AuNPs in locations 1 and 2-4 in 3D (Table S7, columns E, F). The sum of the fractions must be equal to 100%. Inspecting column G of Table S7 we find that there are sums that deviate considerably from 100% (color code of the shaded cells: green … close to 100%, light orange … between 90% and 110%, dark orange … more than 110%). The deviations from 100% mean that the parameters of the MC model do not fit the experimental observations in cryo-TEM images. For in situ loading specimens, there is a good match for parameter t = 7.5 nm for PS-Au5 (Table S7, column G, line 2) and for parameter t = 10 nm for PS-Au10 (Table S7, column G, line 6). But for post loading specimens, there is no acceptable match for any reasonable parameter t (Table S7, column G, lines 7 – 12). Thus the assumption that there is no preference of AuNPs for location 1 or 2 or 3 or 4 in post loading specimens is not valid.

In the second step we modified the MC model for post loading specimens. Cryo-TEM images indicate that in post loading specimens AuNPs prefer location 1. The fractions indicated in Figure 2 (copied into Table S7, columns A, B) are larger for post loading than for in-situ loading. The modified MC model has an additional parameter a expressing the preference of AuNPs to location 1: the fraction of AuNPs in location 1 in 3D is equal to a, 0% < a < 100%. We varied parameter a form 10% to 95%, calculated corresponding correction factors (Figure S9) and looked for best match to 100% criterion (Table S7, column G, lines 13 – 18). For specimen PS-Au5p the best match is a = 70%, for specimen PS-Au10p it is a = 95%.

In conclusion, columns E, F, lines 2, 6, 14, 18 of Table S7 (green shaded cells) give reasonable estimate of fractions of AuNPs in locations 1 and 2+3+4 in 3D (compare to Figure 2 in paper).

2 Background Information

2.1 Composition and function of photo-crosslinked polymersomes

For the creation of photo-crosslinked polymersomes (Figure 1) we used our standard block copolymers (100% of BCP1 or BCP2) consisting of a hydrophilic block made from poly(ethylene glycol) (PEG) and of a hydrophobic block, composed by major pH-sensitive diethylaminoethyl methacrylate (DEAEMA) units and minor UV-crosslinking units, either 4-(3,4-dimethylmaleic imido)butyl methacrylate (DMIBMA) or 2-hydroxy-4-(methacryloyloxy) benzophenone (BMA). The swelling of photo-crosslinked polymersomes in our study is triggered by the protonation of tertiary amino group for DEAMEMA units in the hydrophobic block at acidic pH, switching the polarity of the whole block from hydrophobic to hydrophilic and vice versa (= shrinkage of vesicles), when impermeable membranes are generated at basic environment. As a result the vesicles can shrink and swell upon a pH switch in controlled and repeatable manner[1-3] for the fabrication enzymatic nanoreactors.[5] This is possible due to the UV irradiation of two units of DMIBMA in a [2+2]-photo-dimerization process, while BMA units undergo radical H abstraction process for crosslinking hydrophobic block (= polymersome membrane) (Figure 1 in main text). Parameters of BCPs are presented in Table S2 and S3.

2.2 Principles of AF4

The separation principle of AF4 is based on the different diffusion properties of objects with different sizes (see SI). Coupling AF4 with size sensitive detection as static and dynamic light scattering (Figure S17) we are able to determine the distribution of the radius of gyration, the hydrodynamic radius and the molar mass as well as the conformation and shape of the particles. If concentration sensitive detectors as UV-Vis (Figure S16) or refractive index (RI) detector are used (Figure S18-S22), quantification of different components in the separated system can be performed.

The separation of Asymmetrical Flow Field Flow Fractionation (AF4) takes place in a long and narrow channel, where a carrier liquid transports the molecules. Perpendicular to the main

flow direction a force field will be applied which influences the sample molecules to separate them. Caused by the channel architecture the laminar flow generates a parabolic flow profile with fastest flows in the middle and lowest near the walls of the channel. The vertical force field transports the molecules near the accumulation wall, while the contrary diffusion force shifts the molecules into the faster regions of the flow profile. An exponential concentration profile is formed after equilibrium state of force field and diffusion. Depending on their diffusion coefficient (D) and hydrodynamic diameter (D_h), based on Stokes - Einstein – equation, the molecules will be separated.

Smaller components move into the faster flows farther away from the accumulation wall and therefore elute before large molecules. The separation is caused by different diffusion coefficients by inducing a flow field with a perpendicular liquid flow. A permeable wall (porous frit covered with an ultrafiltration membrane) allows the cross flow to act as force field. The retention time (t_r) is given by following equation:

$$t_r = \frac{w^2 \eta t_0 \pi \dot{V}_c}{2 V_0 k T} d_h$$

where w is the channel height, η is the carrier fluid viscosity, t_0 is the void time, $\dot{V}_c$ is the cross-flow rate, V_0 is the void volume, k is the Boltzmann's constant, and T is the temperature.

2.3 Principles of Static light scattering

For the absolute determination of molar mass the application of a Multi Angle Laser Light Scattering (MALLS) detector in combination with a concentration sensitive detector (RI detector) is essential. After separation, the molecules passed the flow cell and the scattered light will be detected at multiple angles.

In contrary to small polymers, that scatter light isotropically in all directions, sufficiently large molecules (*$d > \lambda/20$, λ* is the wavelength of the applied laser light) scatter light with different intensity under different angles. This is caused by the interference phenomena that occur if there is more than one scattering center per molecule. The scattering intensity R(q) for an angle q is connected with the weight-averaged molar mass (M_w) in the following equation:

$$K^* = \frac{c}{R(q)} = \left[\frac{1}{M_w} + P(q)\right] + A_2 c$$

where c is the polymer concentration, and A_2 is the second viral coefficient. P(q) describes the angular dependency of the scattered light. The optical constant K is defined as:

$$K^* = 4\pi^2 \, n_0^2 \left(\frac{dn}{dc}\right)^2 / (\lambda_0^4 \, N_A)$$

where n_0 the refractive index of the solvent, dn/dc the refractive index increment, λ_0 the wavelength of the laser, and N_A Avogradro`s constant. By plotting $K^* \, c/R(q)$ versus $\sin^2(q/2)$ the interception of the y axis is characterized by molar mass and the radius of gyration (r_g) is given by the slope.

The light scattering intensity is strongly dependent on the particle size which leads to rather low sensitivity for smaller molecules. AF4 in combination with SLS detection with multiple angles enables the possibility to obtain additional information about the conformation and scaling properties and aggregation behavior of the molecules.

2.4 Principles of Dynamic light scattering

Dynamic light scattering (DLS, also known as photon correlation spectroscopy, PCS) is used for the determination of diffusion coefficient (D) and thus the hydrodynamic size of particles.

A laser beam passes through the sample solution where the light gets partially scattered by the particles with certain intensity. Brownian molecular motion of the particles leads to interferences of scattered rays from different molecules. This can be observed as a time-dependent fluctuation of the scattered light. The fluctuation is of higher frequency for smaller particles with higher diffusion coefficients and of lower frequency for larger particles with lower D. For evaluation of these fluctuations, they can be processed into physical parameters by using a correlator producing an autocorrelation function. In principle, the correlator compares the signal (light scattering intensity) at time t with the signal at time (t+τ), where τ is as time increment which is being increased from less than 1 µs up to several seconds. If the signals are similar, the correlator gives a high value, if the signals are more different, the value will be lower. Typically, the correlation is high at short time scales (small τ) and decreases exponentially at larger time scales. For monomodal sample the autocorrelation function can be expressed with an exponential decay function whereas for a multimodal sample a superposition of multiple exponential functions has to be established. The experimental data points need to be fitted by theoretical curves that can be expressed by:

$$g(\tau) = 1 + \beta \, e^{(-2D_q \, 2\tau)}$$

where $g(\tau)$ is the correlation function, β the amplitude of the function, D the diffusion coefficient, q the scattering vector (dependent on refractive index of solution, wavelength of the applied laser light, and the observed scattering angle) and τ the time increment.

Making use of the Stokes-Einstein relation, the diffusion coefficient can be converted into the hydrodynamic diameter:

$$d_h = \frac{k_B \, T}{3\pi \, \eta \, D}$$

Besides d_h as the hydrodynamic diameter and D as the diffusion coefficient, the equation contains Boltzmann's constants k_B, the temperature T, and the solvent viscosity.

In contrast to SLS, DLS allows analysis of smaller particles. For the characterization of mixtures with different components, e.g. single molecules and aggregates it is necessary to perform a previous separation. Otherwise only an average value will be obtained.

3 Additional Figures and Tables

3.1 Size, molar mass and zeta-potential of the used cargo nanoparticles and (bio)macromolecules

Table S1: Characterisation of the used cargo molecules Myoglobin, Esterase and PEI-precursors (before labelling with Rhodamin B) as well as of the AuNPs.

Substance	Diameter [nm]	Molecular weight [kg/mol]	Mn [kg/mol]	Mw [kg/mol]	PD (Mw/Mn)
	(DLS in batch)	(Literature or elemental analysis)	(by AF4)		
Myoglobin	4.9	17 [ref. 6]	16.3	16.6	1.02
Esterase	9.8	180 [ref. 7]	185	362	1.95
Precursor PEI-5	7.0	15	20.9	23.0	1.1
Precursor PEI-25	14.7	35.5	51.7	111	2.18
AuNP-5	5 [a]	-	-	-	≤ 0.2 [a]
AuNP-10	10 [a]	-	-	-	≤ 0.2 [a]

[a]Diameter and dispersity values as given by Sigma Aldrich.

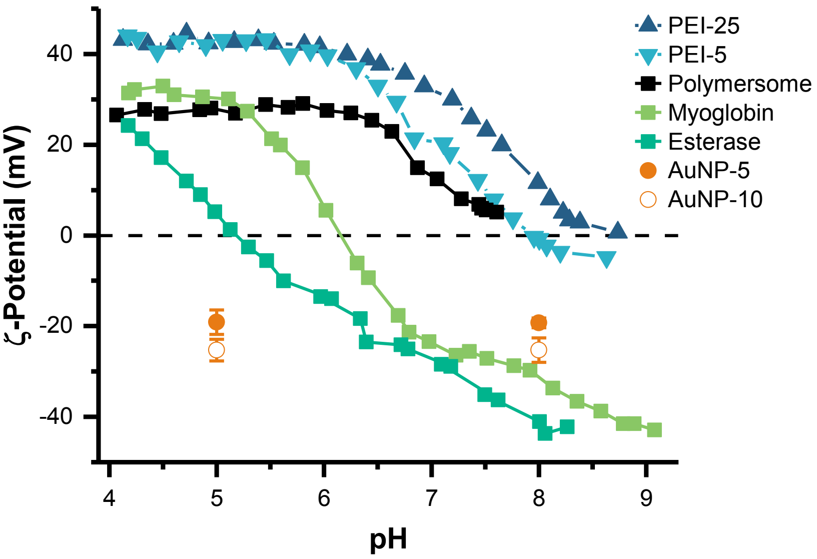

Figure S1: ζ-Potential curves of polymersomes, used glycopolymers PEI-5 and PEI-25, as well as of the used enzymes Myoglobin and Esterase and gold nanoparticles, respectively. Measured at 1 mg/ml in Millipore water.

3.2 Parameters of block copolymers used for polymersome formation

Table S2. Composition, block ratio, molecular weight, dispersity the used block copolymer BCP1 as determined by ^{1}H-NMR and GPC.

Polymer	n_{DEAEMA}[1]	n_{DMIBMA}[1]	M_n (g/mol)[1]	M_w/M_n[2]
**BCP1**	83	21	23 100	1.19

[1] as determined by ^{1}H-NMR-Spectroscopy
[2] as determined by GPC

Table S3. Composition, block ratio, molecular weight, dispersity the used block copolymers BCP2 as determined by ^{1}H-NMR and GPC.

Polymer	n_{DEAEMA}[1]	n_{BMA}[1]	M_n (g/mol)[1]	M_w/M_n[2]
**BCP2**	80	10	20 000	1.31

[1] as determined by ^{1}H-NMR-Spectroscopy
[2] as determined by GPC

3.3 AuNP encapsulation

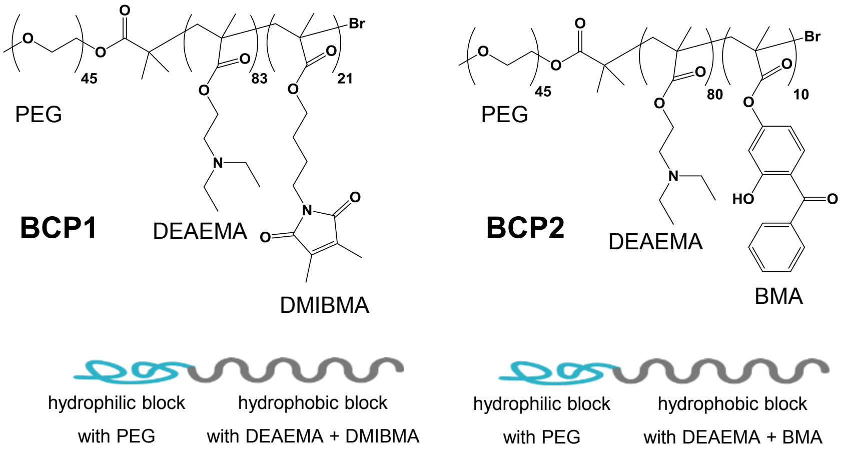

Figure S2: Block copolymer BCP1 used in polymersome formation for further in situ and post encapsulation of nanoparticles, except Au nanoparticles using BCP2 for polymersome formation.

The block copolymer BCP2 used for formation of polymersomes and encapsulation of AuNPs is very similar to BCP1 consisting of DMIBMA [4-(3,4-diemthylmaleic imido)butyl methacrylate] as a crosslinking unit. The only difference is that 2-hydroxy-4-(methacryloyloxy) benzophenone (BMA) has been used as UV-crosslinking unit instead of DMIBMA in BCP2. Polymersomes formed from BCP2 exhibit very similar properties, especially concerning the reversible swelling and shrinking,[3] like the ones made from the first BCP1 system used for protein and protein mimics uptake and release.[2]

Table S4: Specifications of gold nanoparticle encapsulated polymersomes purified via HFF after loading.

Code	Molar Ratio [AuNP:BC]	Method	Diameter[a] [nm]	PDI[b]	Zeta potential [mV] pH8	pH 5
PS1-Au5	3.8	*in situ*	128.2±1.6	0.20	6.6±1.3	22.9±0.4
PS1-Au10	3.8	*in situ*	116.6±1.2	0.24	6.8±0.5	24.2±1.0
PS1-Au5*p*	3.8	*post*	133.9±1.4	0.27	2.5±0.1	17.2±0.3
PS1-10*p*	3.8	*post*	127.4±1.2	0.22	0.1±0.5	21.3±0.7
PS1	-	-	120.0±1.6	0.20	5.0±0.9	23.7±0.4

[a]Hydrodynamic diameter is measured by DLS at pH 8.
[b]PDI = polydispersity of polymersome/gold nanoparticle assemblies that shows the size variation.

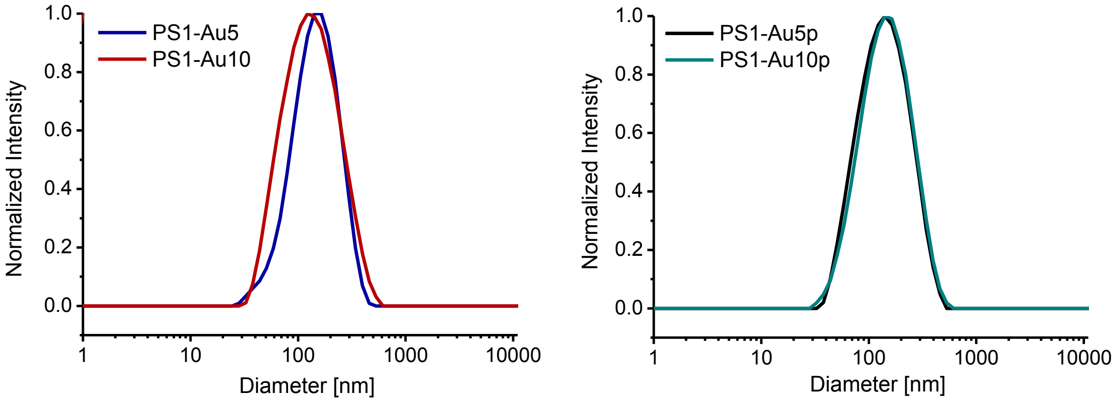

Figure S3: Intensity size distribution of gold nanoparticle loaded polymersomes prepared through in-situ encapsulation, PS1-Au5 and PS1-Au10, at pH 8 (left). Intensity size distribution of gold nanoparticle loaded polymersomes prepared through post-encapsulation, PS1-Au5*p* and PS1-Au10*p*, at pH 8 (right).

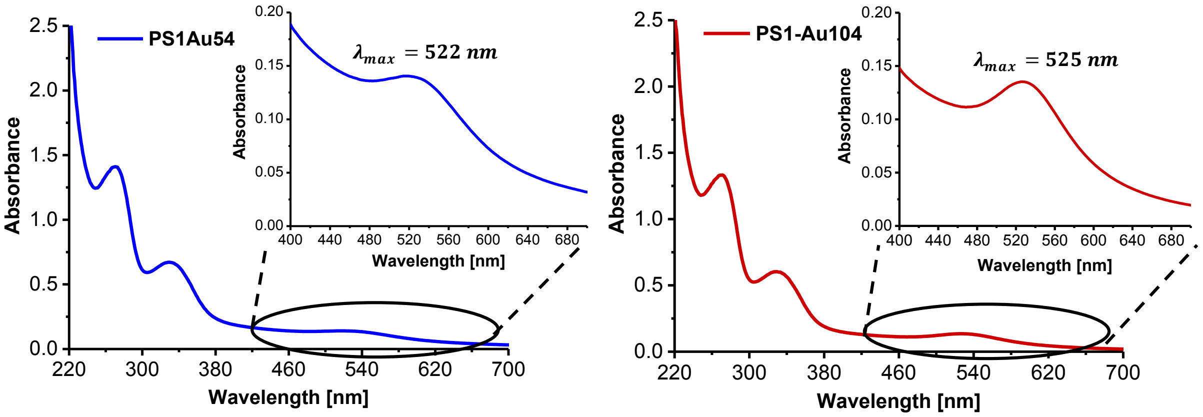

Figure S4: UV-Vis spectra of HFF purified (a) 5 nm and (b) 10 nm sized-gold nanoparticles (AuNP) encapsulated into polymersomes using the in situ method, PS-Au5 and PS1-Au10 respectively. The inserts show the SPR region of the gold nanoparticles.

3.4 Monte Carlo simulation for the determination of AuNPs in three-dimensional confinement of polymersomes

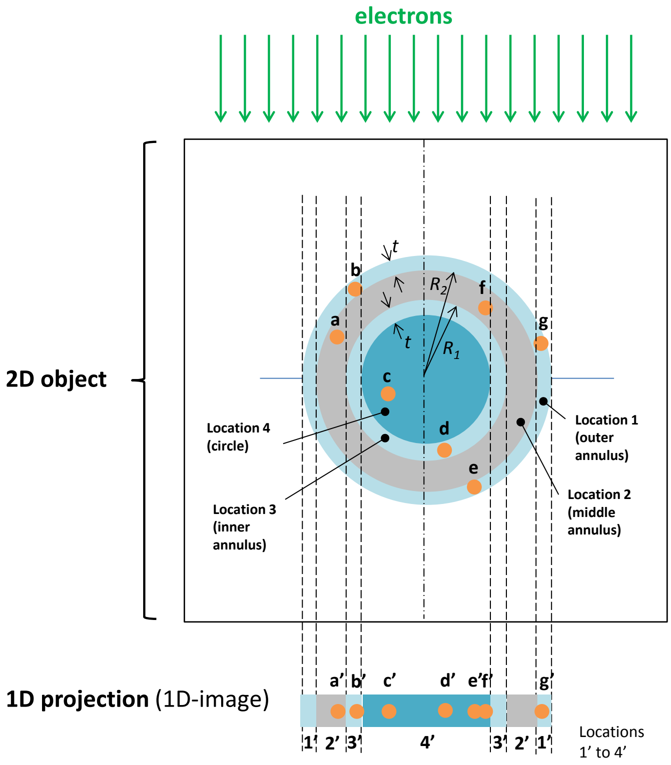

Figure S5: Explanation of the projection effect in electron microscopy: Two-dimensional polymersome loaded with AuNPs projected onto a line, i.e. into a one-dimensional image. In an analogous way, three-dimensional computer model can be projected into a plane (i.e. two-dimensional image).

Table S5: Number of AuNPs in different parts of a polymersome corresponding to **Figure S5**.

	Number of AuNPs in							
	**2D outer shell**	**2D membrane**	**2D inner shell**	**2D lumen**	**1D projection of outer shell**	**1D projection of membrane**	**1D projection of inner shell**	**1D projection of lumen**
	3	2	1	1	1	1	1	4
	Particles {b,g,e}	Particles {a,f}	Particle {d}	Particle {c}	Projection {g'}	Projection {a'}	Projection {b'}	Projection {c',d',e',f'}

Table S6: Number of AuNP in different parts of 10 simulated 2D polymersomes. PS = Polymersome.

	**Number of AuNPs in**							
**PS**	**2D outer shell**	**2D membrane**	**2D inner shell**	**2D lumen**	**1D projection of outer shell**	**1D projection of membrane**	**1D projection of inner shell**	**1D projection of lumen**
1	3	2	1	1	1	1	1	4
2	2	2	1	2	1	0	3	3
3	4	0	2	1	1	3	0	3
4	2	2	0	3	1	1	2	3
5	2	4	0	1	1	3	1	2
6	4	1	1	1	0	2	2	3
7	1	5	1	0	0	4	1	2
8	3	1	2	1	2	2	0	3
9	2	2	3	0	0	2	2	3
10	1	2	2	2	1	1	2	3
**Mean**	**2.40**	**2.10**	**1.30**	**1.20**	**0.80**	**1.90**	**1.40**	**2.90**
**Std.Dev.**	**1.02**	**1.37**	**0.90**	**0.87**	**0.60**	**1.14**	**0.92**	**0.54**

Table S7. Estimation of fraction of AuNPs in locations 1 and 2-4 in 3D (columns E, F) based on fractions of AuNPs in location1 and 2-4 in cryo-TEM images (columns A, B) and on MC models. Suitable MC models (columns C, D) are identified as best fits (column G). (Values in columns A, B are copied form Fig. 2.)

		A	B	C	D	E	F	G	
Sample code	Encaps. method	Fraction of AuNPs: in location 1 (measured in 2D image)	Fraction of AuNPs: in locations 2+3+4 (measured in 2D image)	Particle distribution model	Parameter of the model	Fraction of AuNPs: in location 1 (estimated for 3D reality: based on 2D images and MC model)	Fraction of AuNPs: in locations 2+3+4 (estimated for 3D reality: based on 2D images and MC model)	The sum of fractions (must be 100%)	
PS-Au5	in situ	15.6	84.4	uniformly	*t* = 5 nm	50.2	65.6	115.8	1
					t = 7.5 nm	40.9	60.4	101.3	2
					t = 10 nm	35.8	55.7	91.5	3
PS-Au10	in situ	18.0	82.0	uniformly	*t* = 5 nm	58.4	65.4	123.8	4
					t = 7.5 nm	50.2	60.2	110.4	5
					t = 10 nm	43.4	55.8	99.2	6
PS-Au5p	Post	24.5	75.5	uniformly	*t* = 5 nm	79.5	60.2	139.7	7
					t = 7.5 nm	68.3	55.5	123.8	8
					t = 10 nm	59.0	51.4	110.4	9
PS-Au10p	Post	39.0	61.0	uniformly	*t* = 5 nm	131.9	49.3	181.2	10
					t = 7.5 nm	113.6	45.2	158.8	11
					t = 10 nm	97.7	42.7	140.4	12
PS-Au5p	Post	24.5	75.5	preferably in location 1, *t* = 7.5 nm	*a* = 60 %	66.7	38.9	105.6	13
					a = 70 %	67.3	31.0	98.3	14
					a = 80 %	66.8	21.5	88.3	15
PS-Au10p	Post	39.0	61.0	preferably in location 1, *t* = 10 nm	*a* = 80 %	96.7	18.5	115.1	16
					a = 90 %	97.3	10.0	107.2	17
					a = 95 %	94.0	5.6	99.6	18

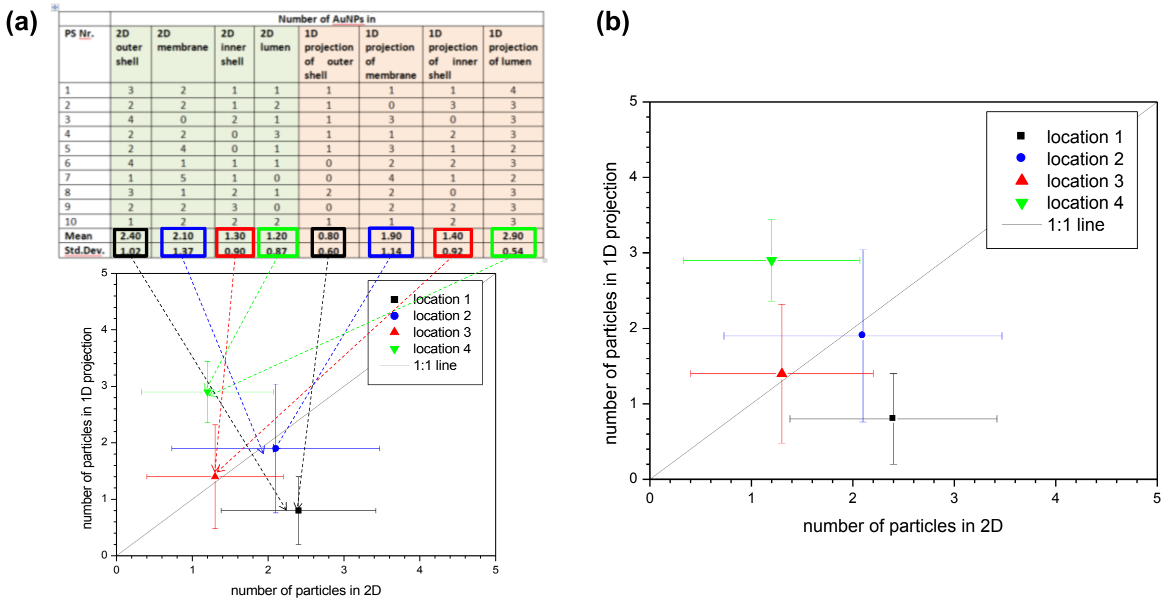

	Number of AuNPs in							
PS Nr.	2D outer shell	2D membrane	2D inner shell	2D lumen	1D projection of outer shell	1D projection of membrane	1D projection of inner shell	1D projection of lumen
1	3	2	1	1	1	1	1	4
2	2	2	1	2	1	0	3	3
3	4	0	2	1	1	3	0	3
4	2	2	0	3	1	1	2	3
5	2	4	0	1	1	3	1	2
6	4	1	1	1	0	2	2	3
7	1	5	1	0	0	4	1	2
8	3	1	2	1	2	2	0	3
9	2	2	3	0	0	2	2	3
10	1	2	2	2	1	1	2	3
Mean	2.40	2.10	1.30	1.20	0.80	1.90	1.40	2.90
Std.Dev.	1.02	1.37	0.90	0.87	0.60	1.14	0.92	0.54

Figure S6: Illustration how plots of Monte Carlo simulations are built. PS = polymersome (a). Graphical representation of results (means and standard deviations) from **Table S4**. Relation between apparent locations in 1D image of polymersomes and actual locations in 2D polymersomes (b).

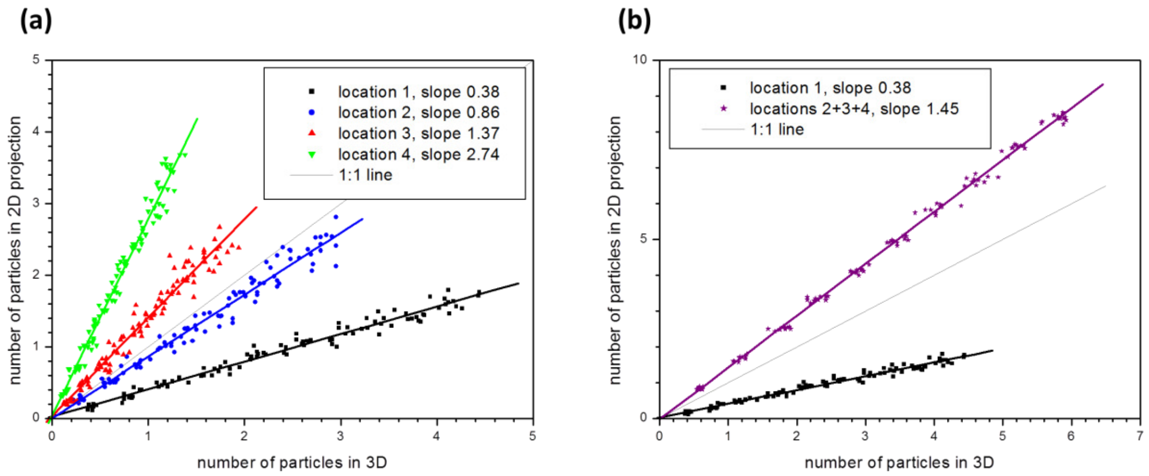

Figure S7: Plot of apparent number of the AuNPs in 2D against the true number of the AuNPs in 3D in the locations 1-4. The slopes of the fitted lines give the correction factors that have to be applied for AuNPs counts in 2D cryo-TEM images to correct them for counts in 3D. (a) Plots for locations 1, 2, 3, 4 individually, (b) Plot for location 1 and sum of particles in locations 2, 3, 4. (For parameters of the simulation see the text.)

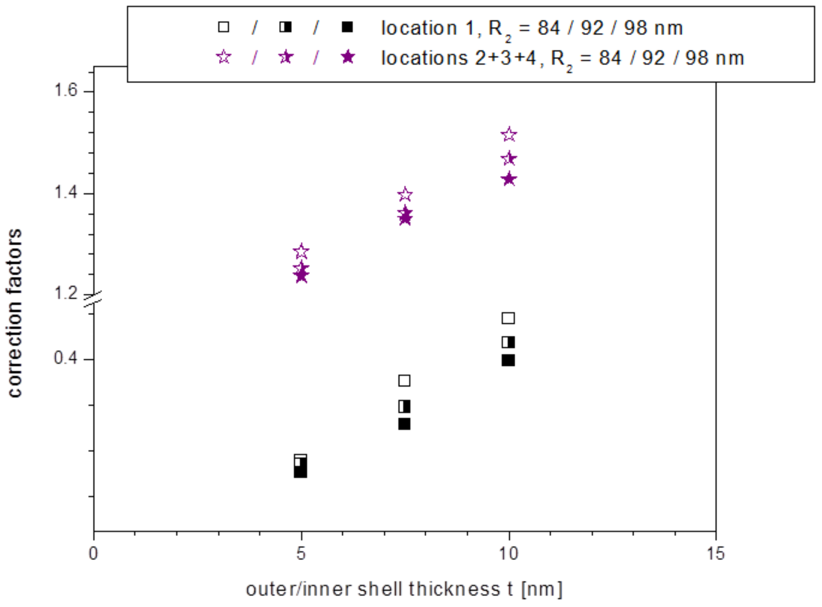

Figure S8: Correction factors for AuNPs fractions as a function of simulation parameter t for polmyersome sizes of 84 nm, 92 nm, 98 nm.

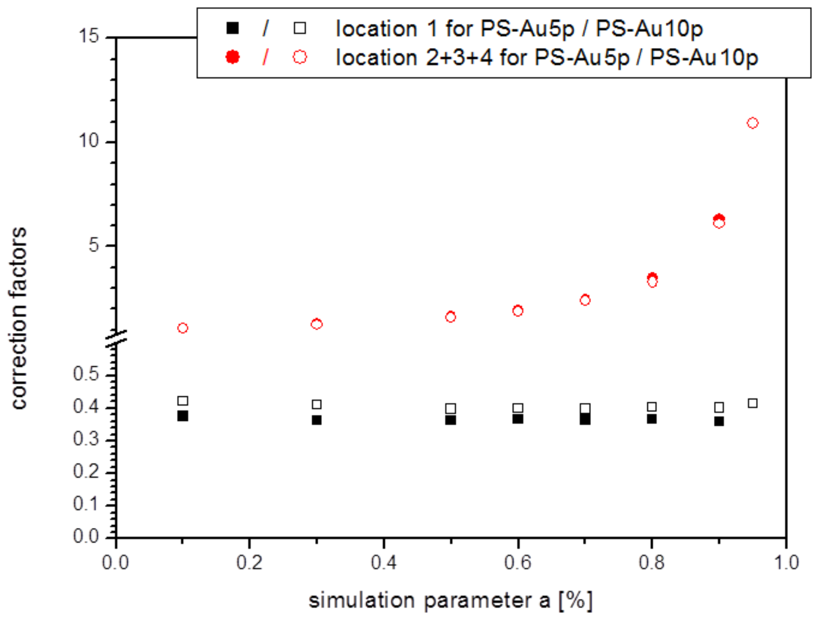

Figure S9: Correction factors for AuNPs fractions as a function of simulation parameter a for polmyersomes in specimens PS-Au5p and PS-Au10p.

3.5 Uptake and release of PEI glycopolymers

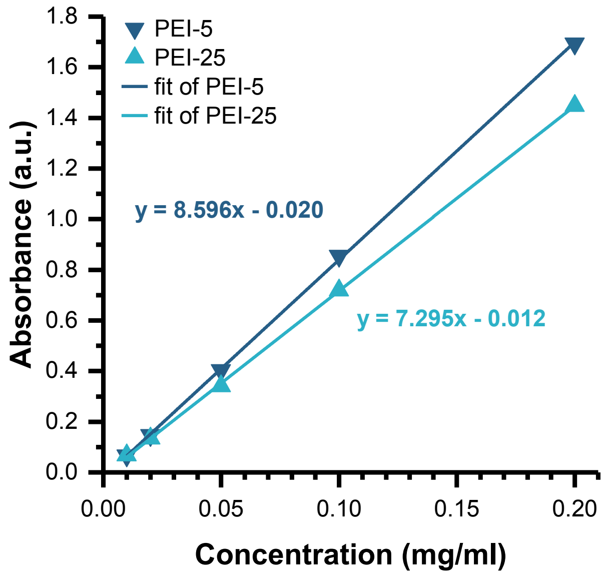

Figure S10: UV-Vis calibration of PEI-5 and PEI-25 glycopolymer concentration against absorbance at 555 nm.

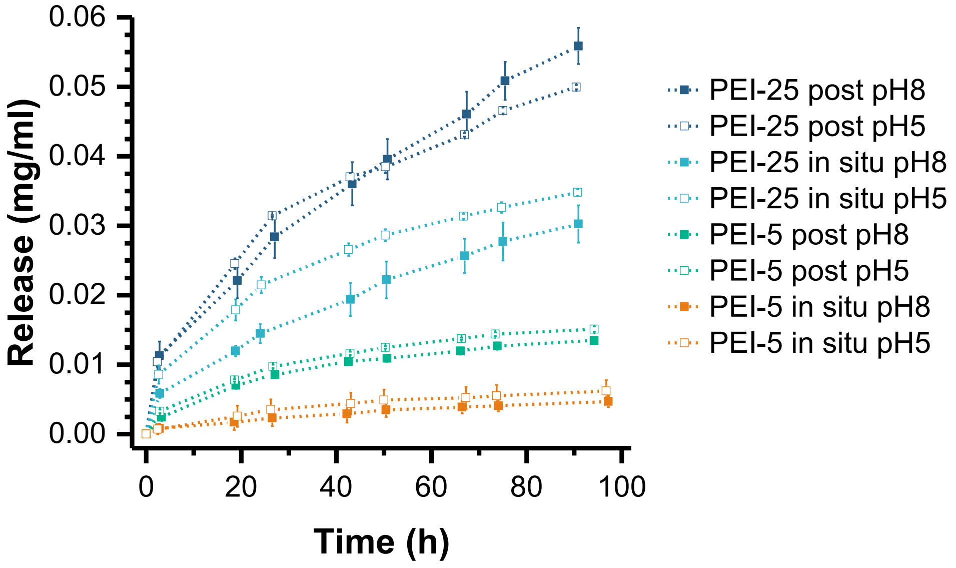

Figure S11: Release kinetics of PEI from polymersomes at pH 5 and 8 given in absolute concentrations in mg/ml.

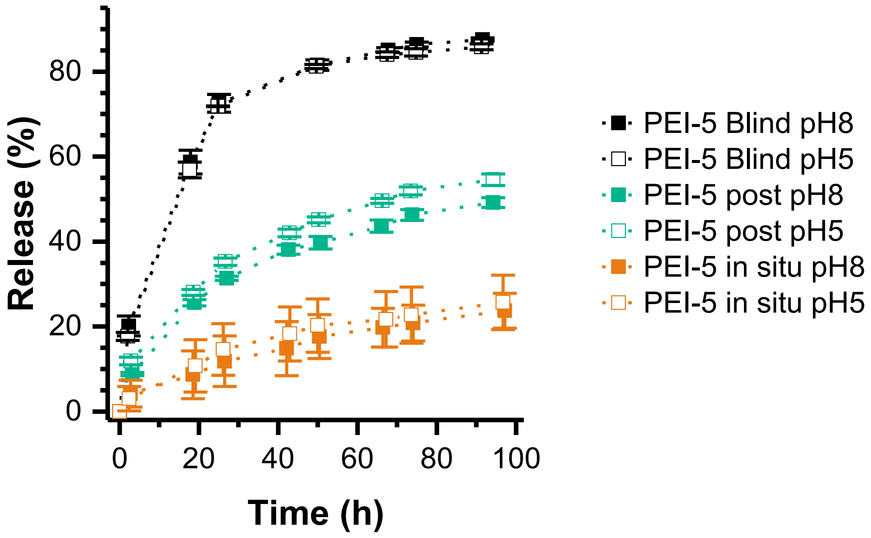

Figure S12: Release kinetics of PEI-5 from polymersomes given as percentage of released mass of originally encapsulated mass. For blind samples simple PEI solution without polymersomes in dialysis tube was used compared to PEI-loaded polymersome in dialysis tube.

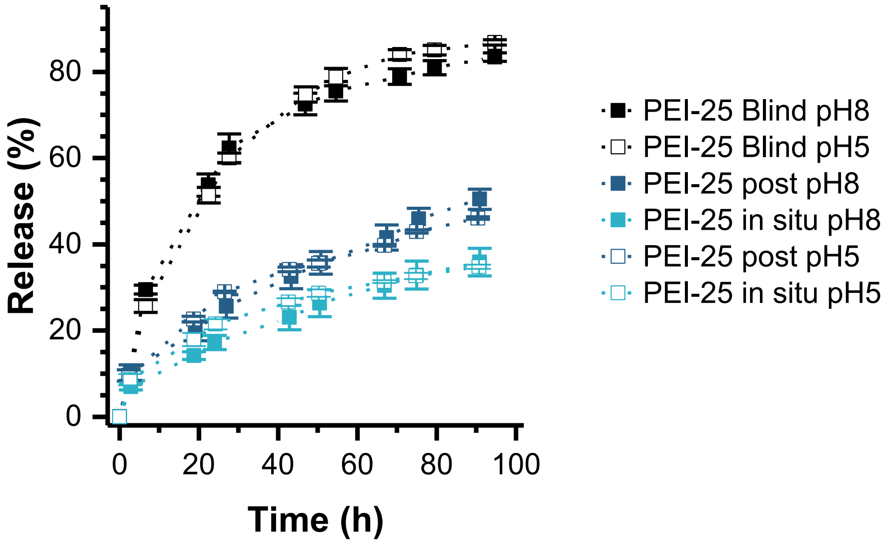

Figure S13: Release kinetics of PEI-25 from polymersomes given as percentage of released mass of originally encapsulated. For blind samples simple PEI solution without polymersomes in dialysis tube was used compared to PEI-loaded polymersome in dialysis tube.

3.6 Polymersome stability against enzymes

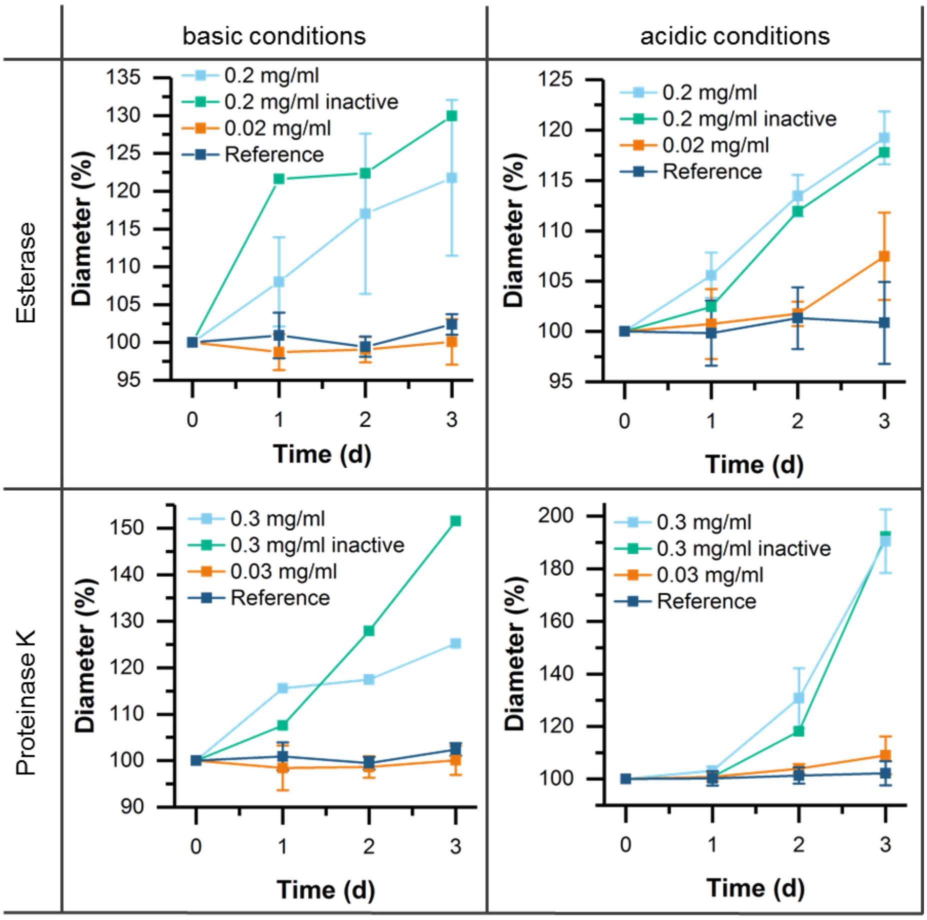

Figure S14: Stability of polymersomes against the digesting enzymes Esterase and Proteinase K at acidic conditions and basic conditions. Vesicle diameters are determined by DLS and normalised to 100 % at t=0.

We incubated our vesicles together with Esterase or Proteinase~K for up to three days at acidic or basic conditions and measured the diameter by DLS. For lower concentration of the enzymes (0.02 and 0.03 mg/ml, respectively) only very minor changes were observed, showing that the integrity of the vesicles is not affected. In case of a 10-fold increase in enzyme concentration (0.2 and 0.3 mg/ml, respectively) the polymersomes showed a significant increase in size after two to three days of storage. We can conclude that our polymersomes are robust and stable against the commonly used digesting enzymes Esterase and Proteinase~K.

Esterase assay. A method of Kilcawley et al. was modified and used to determine Esterase activity.[8] In short, assays are typically conducted in 925 µl PBS at pH 7.4 adding 20 µl of an aqueous solution of Triton X-100 (Sigma-Aldrich, 10 w%), 5 µl of Esterase sample (estimated concentration of 0.2 mg/ml) and 50 µl of p-Nitrophenylacetate (Sigma-Aldrich)

solution in ethanol (c = 10mM). After addition of the substrate, the mixture is rigorously shaken and incubated for 30 s, followed by measuring the absorbance at 405 nm every 10 s for 120 s.

Proteinase K assay. To determine the activity of proteinase K the method of Charney et al. was used with modifications.[9] A sample volume of 0.5 ml is incubated under stirring with 0.5 ml of an azocasein solution (Sigma Aldrich, 2.5w% in PBS) for 30 min at room temperature followed by the addition of 0.5 ml of perchloric acid (1.5 M). After 5 min of incubation the solution is filtered from the formed precipitate using a syringe filter (cellulose-ester, 0.2 µm). 0.3 ml of the solution are diluted with 0.4 ml of PBS and 0.3 ml of NaOH (1 M) and transferred to a cuvette to measure absorption at 420 nm. A blind sample without any enzymes is prepared as blank for the UV/Vis measurements. All samples were measured in triplicates. 1 Unit is defined as conversion of 1 µmol substrate per minute.

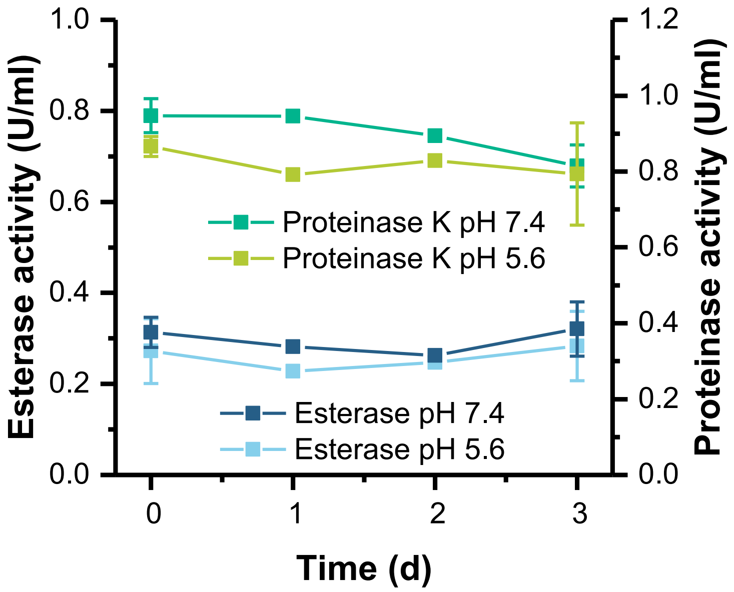

Figure S15: Enzymatic activity of Esterase and Proteinase K in basic and acidic conditions over 3 days while stored together with polymersomes.

3.7 AF4 investigations

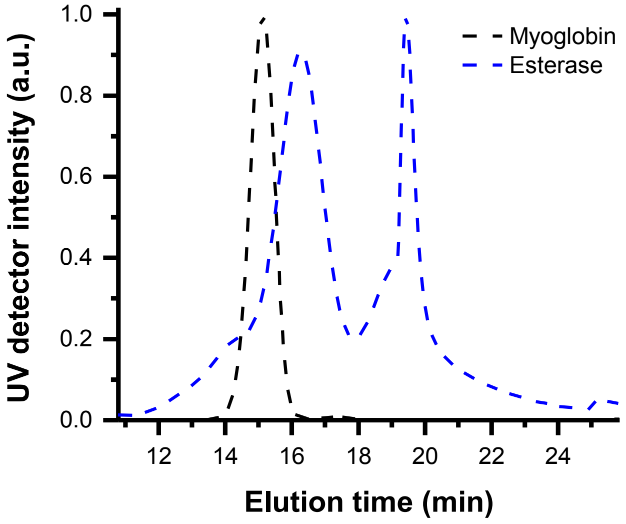

Figure S16: UV Signal of myoglobin (black line, at 410 nm, separation method B) and esterase (blue line, at 275 nm, separation method A) after AF4 separation. For molar masses and molar mass distributions data are presented in **Table S1**.

3.7.1 Empty polymersomes

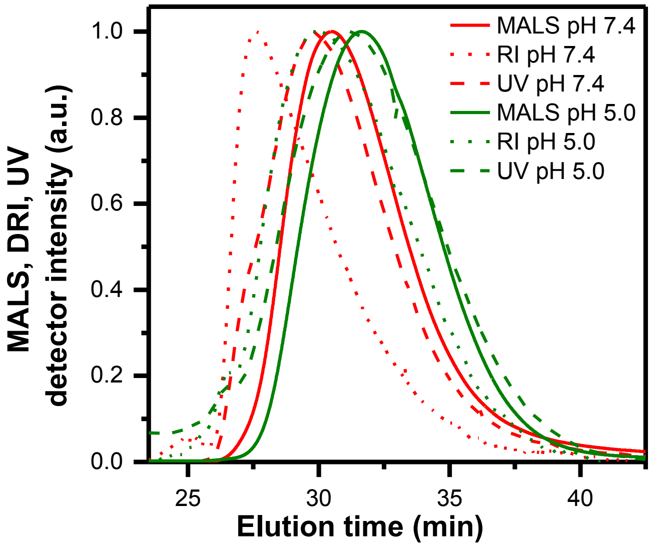

Figure S17: Static light scattering, refractive index and UV signal of polymersomes measured at pH 5 (green lines) and pH 7.4 (red lines) after AF4 separation (method A).

3.7.2 Separation of polymersome and enzymes

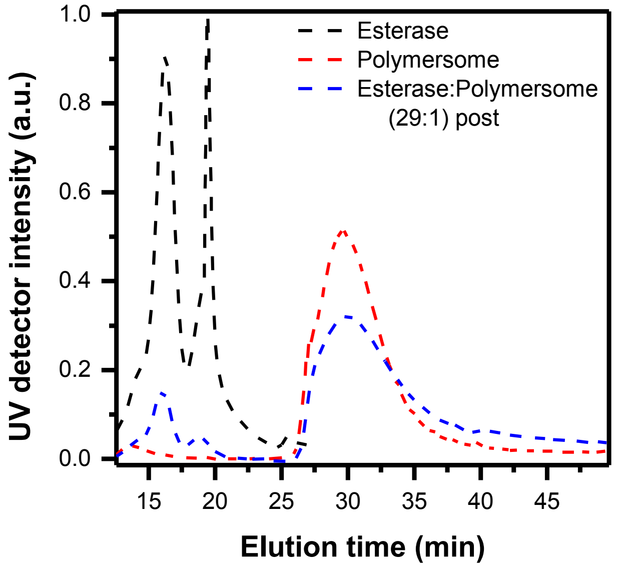

Figure S18: UV signal of polymersome (red line), esterase (black line) and polymersome loaded with esterase (blue line) shows that free esterase can be well separated from the vesicles for further quantification studies. The measurements (separation method A) were performed at 275 nm at which the polymersome and the esterase absorb.

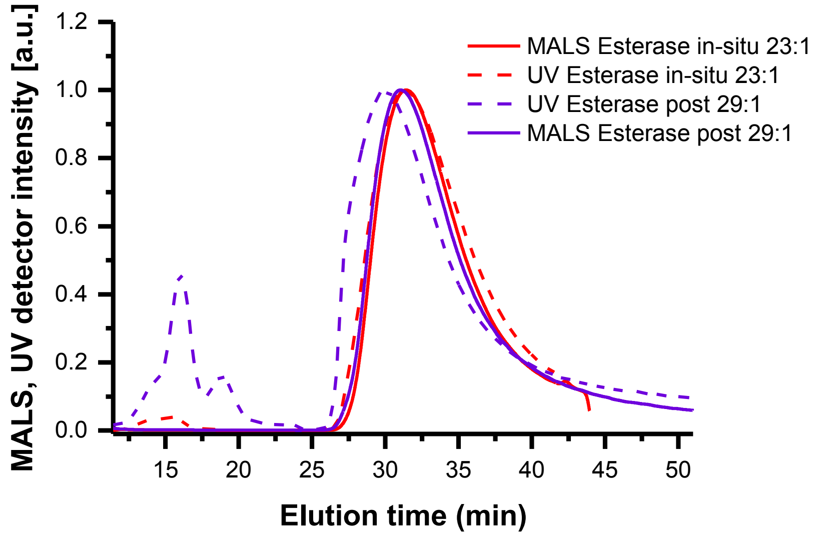

Figure S19: Static light scattering and UV signal (275 nm) of polymersome loaded with esterase in ratio 23:1 (red lines) and 29:1 (violet lines).

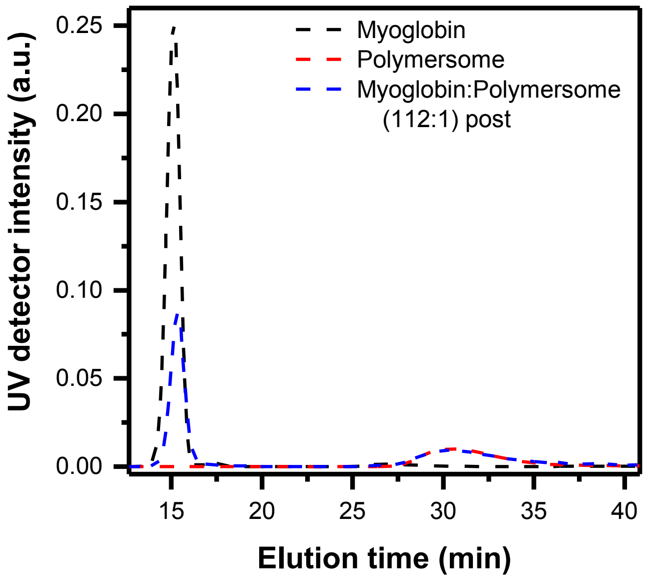

Figure 20: UV signals of polymersomes (red line) , myoglobin (black line) and polymersome loaded with myoglobin (blue line) show that free myoglobin can be well separated from the vesicles and detected via UV at 410 nm for further quantification studies.

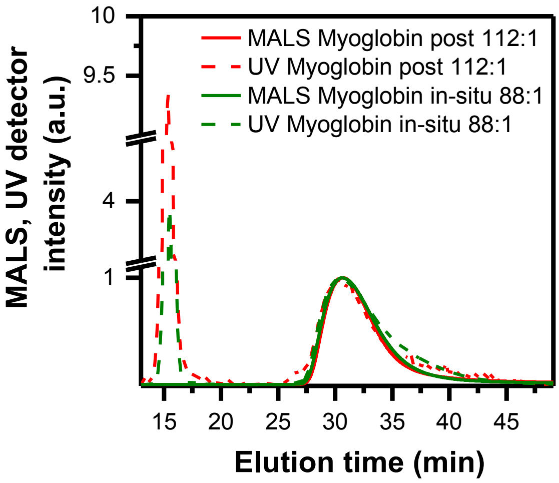

Figure S21: Static light scattering and UV signal (410 nm) of polymersomes loaded with myoglobin in ratio 112:1 (red lines) and 88:1 (green lines) with separation method B.

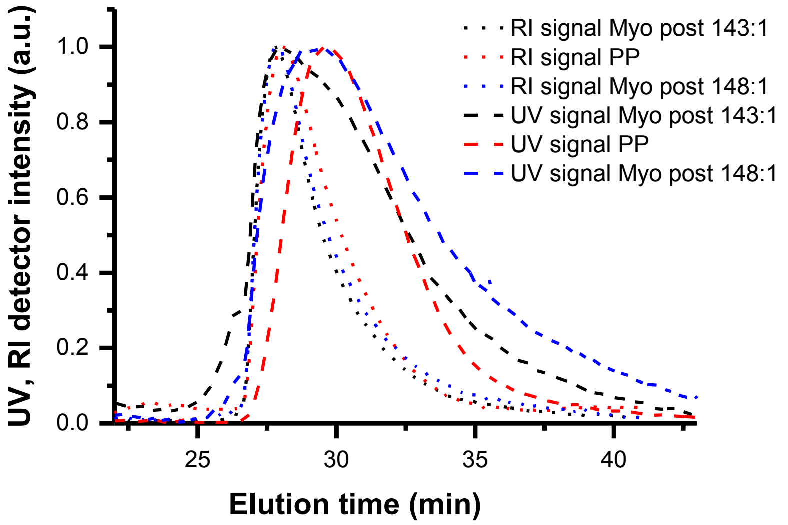

Figure S22: RI and UV signal (410 nm) of polymersome (red lines) and polymersome loaded with myoglobin in ratio 143:1 (black lines) and 148:1 (blue lines) with separation method B.

3.7.3 Polymersome loaded with Esterase - in situ encapsulation

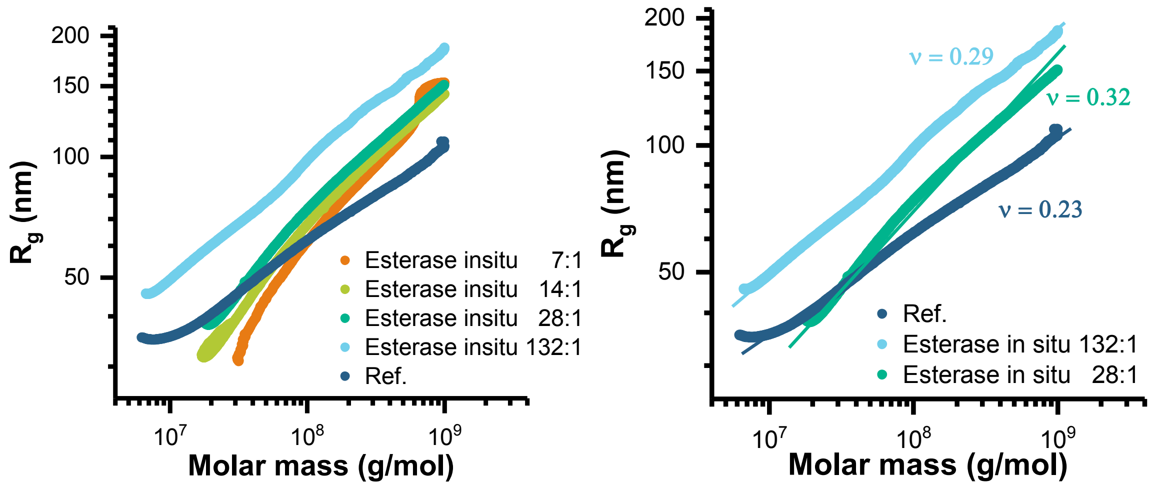

Figure S23: Conformation plot of a pure polymersome (Ref.) and polymersomes loaded with esterase at different ratios. After loading the slope is increased indicating less collapsed membranes and rougher membrane surfaces than for the pure polymersomes.

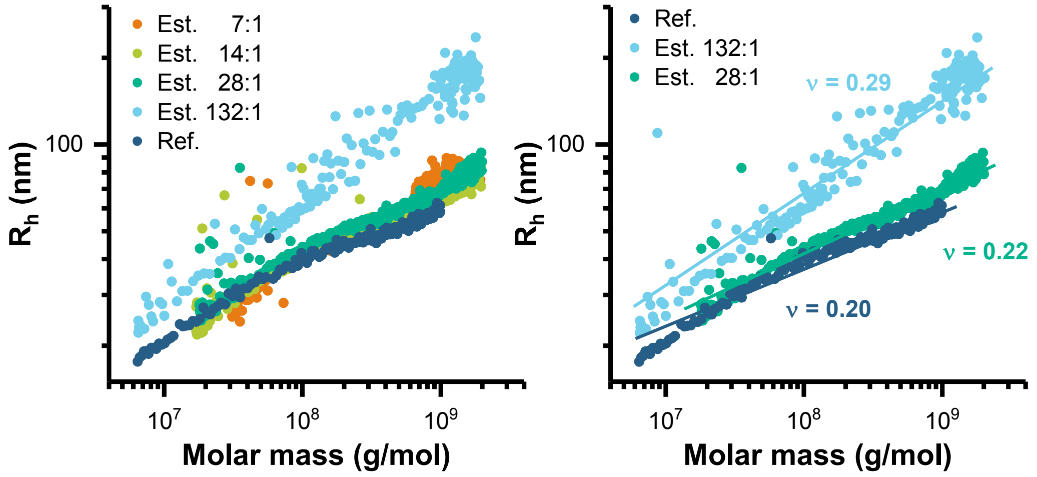

Figure S24: Dependence of the hydrodynamic radius R_h from the molar mass in analogy to the conformational plot based on the radius of gyration R_g.

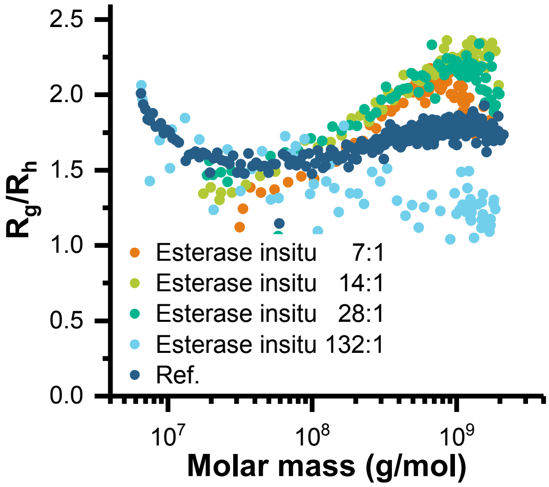

Figure S25: R_g/R_h dependence on the molar mass of a pure polymersome (Ref.) and polymersomes loaded with Esterase in different ratios showing slightly higher ratios after loading corresponding to the lower density of the polymersomes after encapsulation (Figure 27). The R_g/R_h ratio is a parameter between 0.78 for hard sphere and 1.6 for a well rinsed macromolecule.

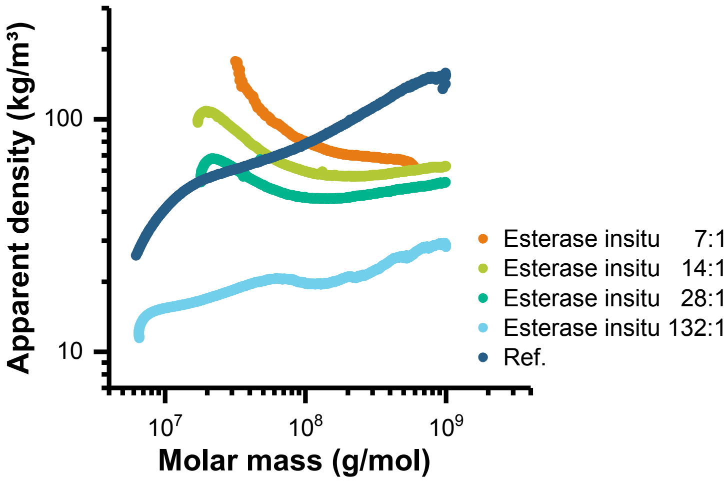

Figure S26: Apparent density calculated according to $\varrho_{app.} = M_w/V(R)$ of a pure polymersome (Ref.) and polymersomes loaded with Esterase in different ratios indicating decrease of the density of the polymersomes after encapsulation.

3.7.4 Polymersome loaded with Esterase - post encapsulation

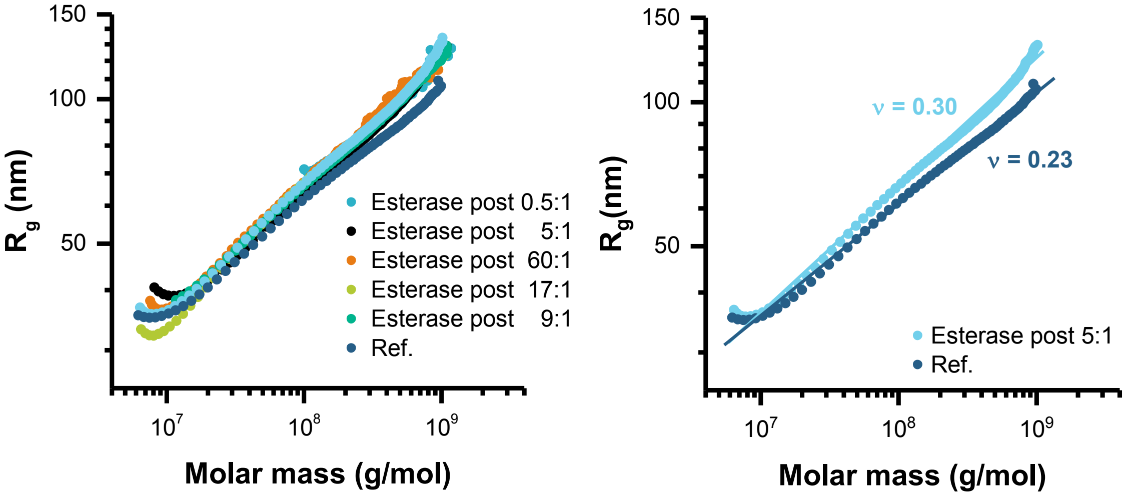

Figure S27: Conformation plot of a pure polymersome (Ref.) and polymersomes loaded with esterase at different ratios. After loading the slope is increased indicating rather less collapsed membranes and rougher membrane surface than for the pure polymersomes.

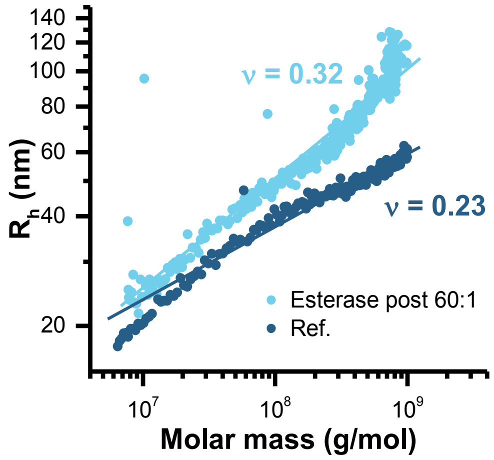

Figure S28: Dependence of the hydrodynamic radius R_h from the molar mass in analogy to the conformational plot based on the radius of gyration R_g.

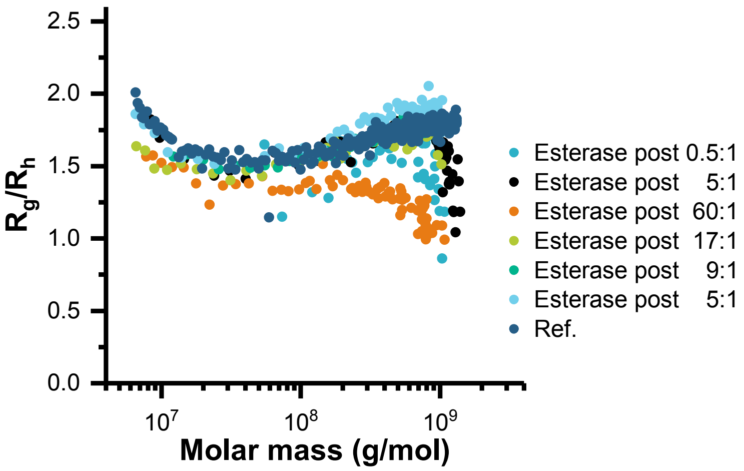

Figure S29: R_g/R_h dependence on the molar mass of pure polymersomes (Ref.) and polymersomes loaded with Esterase in different ratios showing constant shape over the molar mass corresponding to a shape less dense than a hard sphere.

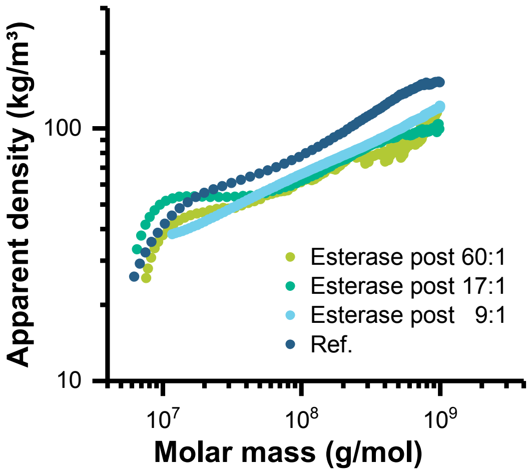

Figure S30: Apparent density calculated according to $\varrho_{app.} = M_w/V(R)$ of pure polymersomes (Ref.) and polymersomes loaded with Esterase in different ratios indicating decrease of the density of the polymersomes after encapsulation.

3.7.5 Polymersome loaded with Myoglobin – in situ encapsulation

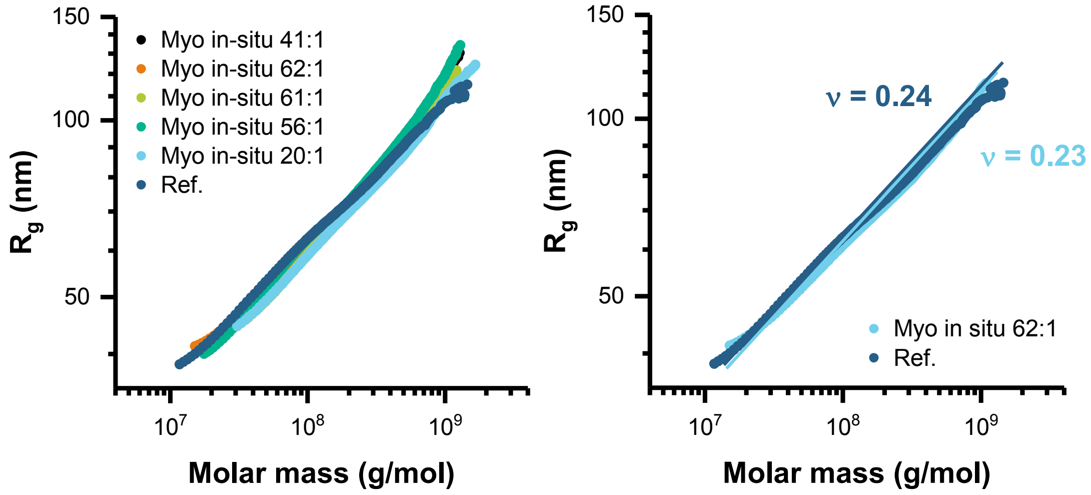

Figure S31: Conformation plot of pure polymersomes (Ref.) and polymersomes loaded with myoglobin at different ratios. No significant influence on the polymersome shape can be indicated after encapsulation.

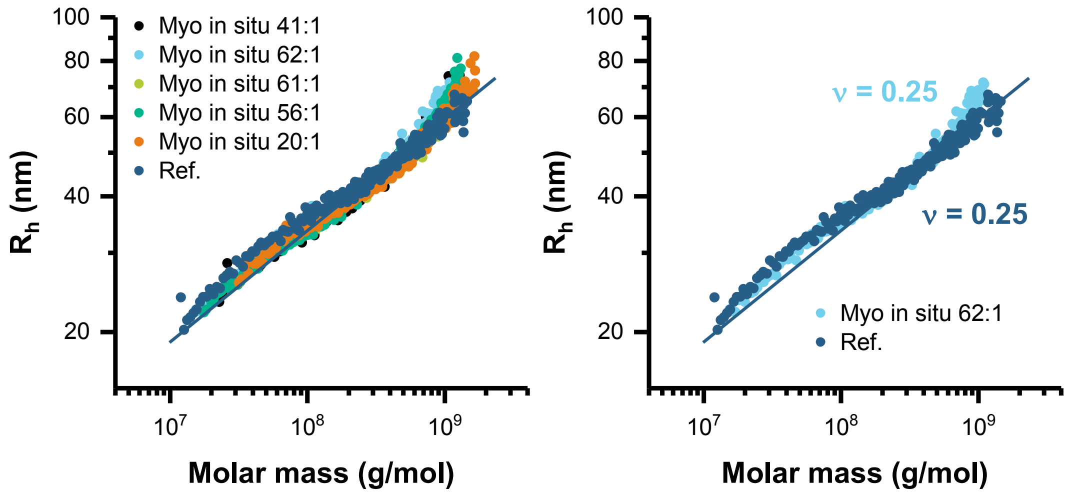

Figure S32: Dependence of the hydrodynamic radius R_h from the molar mass in analogy to the conformational plot based on the radius of gyration R_g. No significant influence on the polymersome shape can be indicated after encapsulation.

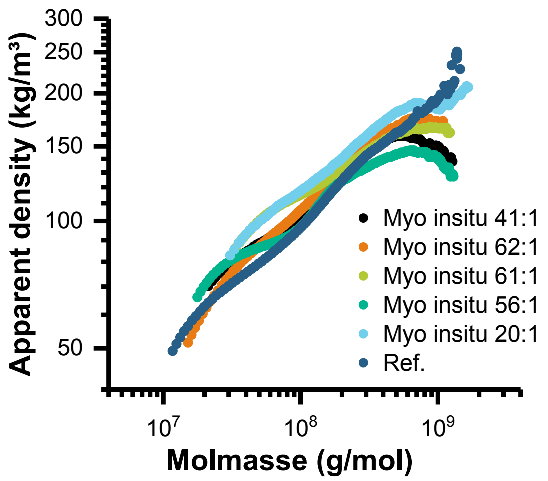

Figure S33: Apparent density calculated according to $\varrho_{app.} = M_w/V(R)$. of pure polymersomes (Ref.) and polymersomes loaded with myoglobin in different ratios. No significant influence on the polymersome shape can be indicated after encapsulation.

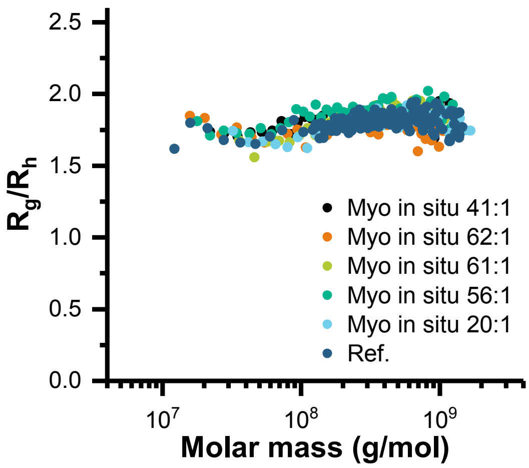

Figure S34: R_g/R_h dependence on the molar mass of pure polymersomes (Ref.) and polymersomes loaded with myoglobin in different ratios showing constant shape over the molar mass corresponding to a shape less dense than a hard sphere.

3.7.6 Polymersome loaded with Myoglobin - post encapsulation

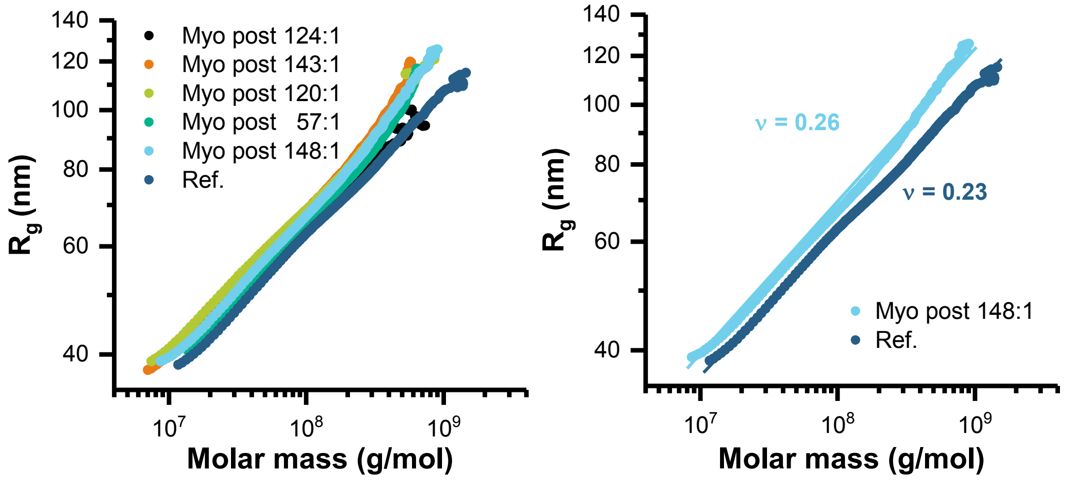

Figure 35: Conformation plot of pure polymersomes (Ref.) and polymersomes loaded with myoglobin at different ratios. After loading the slope is slightly increased indicating rather less collapsed membranes and rougher membrane surface than for the pure polymersomes.

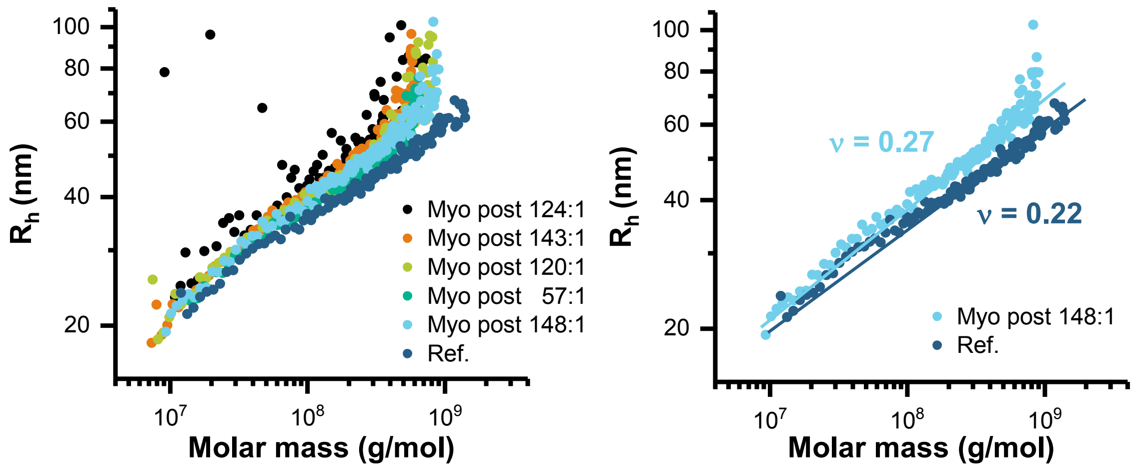

Figure S36: Dependence of the hydrodynamic radius R_h from the molar mass in analogy to the conformational plot based on the radius of gyration R_g.

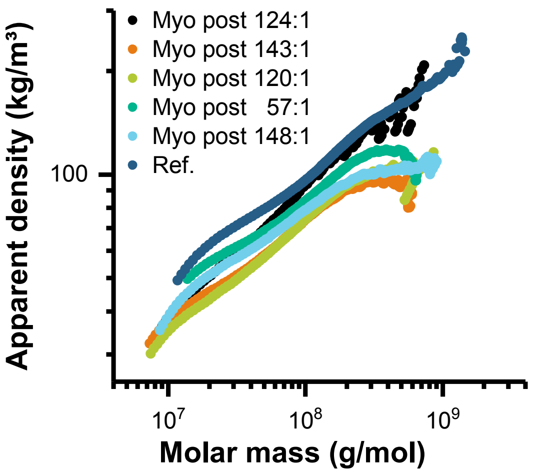

Figure S38: Apparent density calculated according to $\varrho_{app.} = M_w/V(R)$ of pure polymersomes (Ref.) and polymersomes loaded with myoglobin in different ratios indicating slight decrease of the density of the polymersomes after encapsulation.

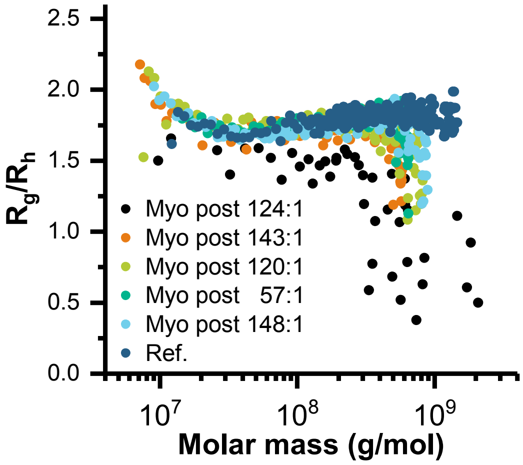

Figure S39: R_g/R_h dependence on the molar mass of pure polymersomes (Ref.) and polymersomes loaded with myoglobin in different ratios showing constant shape over the molar mass corresponding to a shape less dense than a hard sphere.

3.7.7 Quantification of loading capacity by AF4

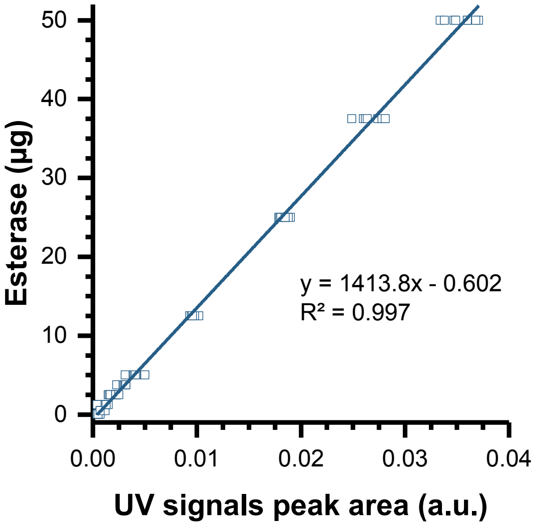

Figure S40a: Calibration curve with peak areas of UV-signal at 275 nm for quantification of esterase using AF4 (separation method A).

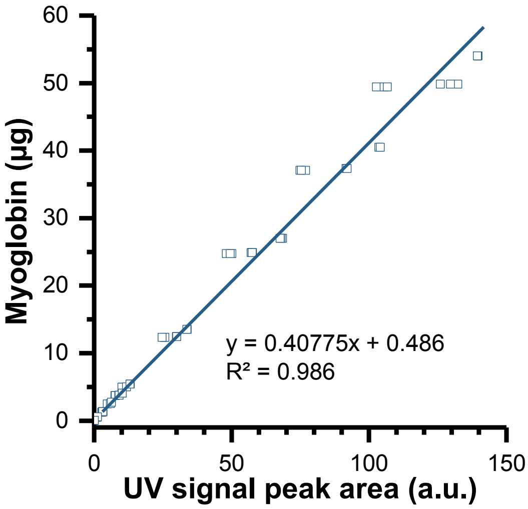

Figure S40b: Calibration curve with peak areas of UV-signal at 410 nm for quantification of myoglobin using AF4 (separation method B).

Table S8: Overview about the quantification results of AF4 studies, encapsulation capacity of polymersomes after exposure to esterase and myoglobin using the post or in situ approach and different polymersome/ protein ratios.

	Enzyme per polymersome in batch,	Isolated (free) enzyme	Enzyme per polymersome Loaded	Enzyme per polymersome (loaded, %)
	number of enzyme molecules per one polymersome			
Esterase *in situ*	8.4	1.1 ±0.1	7.3 ±0.7	86.9 ±8.3
	16.7	2.7 ±0.3	14.0 ±1.4	83.8 ±8.4
	33.6	5.3 ±0.2	28.3 ±1.1	84.2 ±3.3
	167.8	35.8 ±1.9	132.0 ±1.9	78.7 ±1.1
Esterase *post*	3.3	2.8 ±0.6	0.5 ±0.1	15.2 ±3.0
	17.3	12.4 ±1.5	4.9 ±0.6	28.3 ±3.5
	24.8	19.4 ±3.5	5.4 ±1.0	21.8 ±4.0
	34.7	27.1 ±6.4	7.6 ±1.8	21.9 ±5.2
	57.8	40.5 ±1.9	17.3 ±0.8	29.9 ±1.4
	172.8	121.1 ±33.9	51.7 ±14.5	29.9 ±8.4
Myoglobin *in situ*	57.0	37.2 ±3.4	19.8 ±1.8	34.7 ±3.2
	114.0	72.7 ±3.3	41.3 ±1.9	36.2 ±1.7
	151.9	90.2 ±2.0	61.7 ±1.4	40.6 ±0.9
	285.0	224.0 ±1.5	61.0 ±0.4	21.4 ±0.1
	380.1	323.7 ±1.1	56.4 ±0.2	14.8 ±0.1
Myoglobin *post*	76.7	20 ±1.0	56.7 ±2.8	73.9 ±3.6
	268.6	120.2 ±3.5	148.4 ±4.3	55.2 ±1.6
	575.6	432.7 ±8.2	142.9 ±2.7	24.8 ±0.5
	767.4	640.7 ±14.4	120.4 ±5.1	15.9 ±0.7
	959.3	835.5 ±104.6	123.8 ±15.5	12.9 ±1.6

3.8 Cryo-TEM

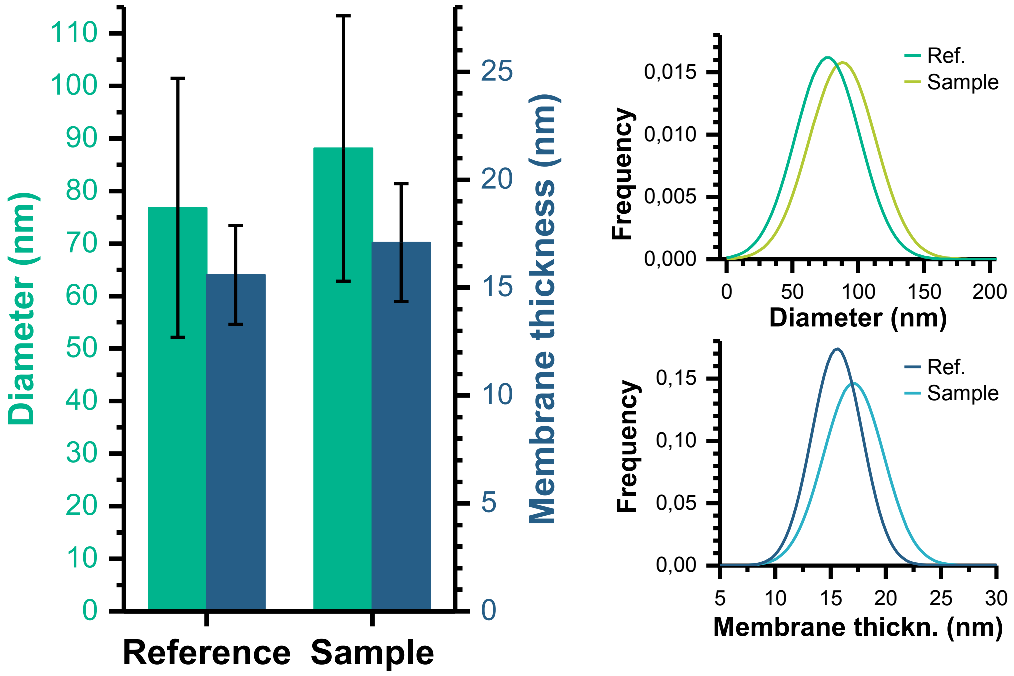

Figure S41: Post encapsulation of Esterase; measurement of diameter and membrane thickness by cryo-TEM. Reference is treated equally (crosslinking, swelling, stirring overnight) but without addition of enzyms. Error bars refer to the standard distribution of the measured lengths (left diagram). Conversion of the obtained values and distributions into Gaussian curves (right diagrams).

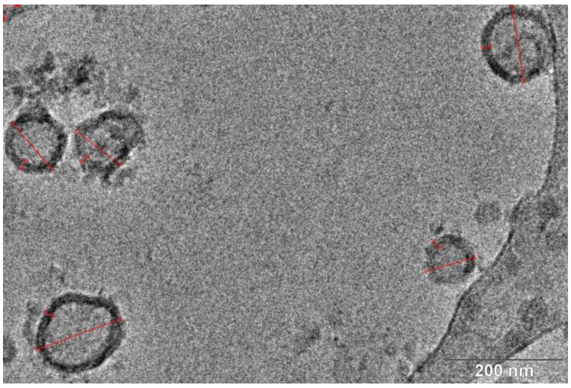

Figure S42: Example of cryo-TEM measurement of diameter and membrane thickness after and before post loading of the polymersome with esterase.

For the investigation of interactions between polymersomes and esterase by cryo-TEM, the enzyme was post-loaded into the vesicles (0.5 mg/ml) as described in the SI (see Myoglobin and Esterase encapsulation for AF4 separation). For the TEM experiments a concentration of

0.1 mg/ml esterase during the loading process was used. As reference a different aliquot of the vesicle solution was treated equally, but instead of Esterase solution only the corresponding buffer was added. After readjusting the pH to 7.4 both samples (vesicles with post-encapsulated Esterase and reference without Esterase) were analysed by cryo-TEM. For each of the samples three grids were prepared. From each grid two different spots were chosen, from each of which images with different magnifications were taken and analysed. This procedure finally resulted in >700 measurements of polymersome's diameters per sample. Because only images of high magnification (>16k) could be used to determine the membrane thickness with the necessary precision and not all investigated spots yielded quality images at very high magnifications, the number of measurements of the membrane thickness is substantially lower, but still includes over 50 membrane measurements per sample. To illustrate the nature of the measurements a representative excerpt of one of the (high magnification) images is given in Figure S44.

3.9 Uptake and release of Myoglobin by/from polymersome

HFF purified polymersomes after myoglobin encapsulation show in contrast to the AF4 studies that the in situ loading is more efficient. To correctly interpret this behaviour it is necessary to also compare the purification conditions with those used for PEI-5 and PEI-25, which show the opposite behaviour (Figure 3, paper). For Myoglobin HFF was conducted using a transmembrane pressure of ca. 180 mbar, whereas for removal of free dendritic glycopolymers PEI-5 and PEI-25 only 120 mbar were applied. The transmembrane pressure can be seen as a measure of the shear forces applied during HFF. As we already showed for PEI the post loading approach is less specific resulting in more encapsulation at location 1. Unfortunately, stronger shear forces are coupled with higher separation power and thus, cargo from these locations is removed quicker than particles at locations 3 or 4. Due to this reasons enhancing the pressure during HFF led to higher encapsulation efficiency for in situ strategy because of its higher selectivity towards the lumen encapsulation.

In summary, after HFF purification at high pressure post encapsulation of myoglobin is less effective, which again confirms the advantage of the higher selectivity towards the lumen (location 4) of the in situ approach. However, both approaches can be used to fabricate an on and off-switchable bionanoreactors.

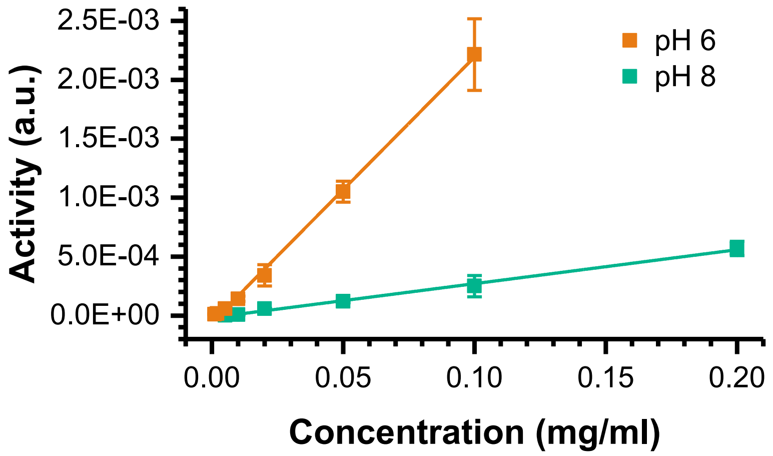

Figure S43: Calibration of Myoglobin activity, the enzyme is dissolved, stored for four days at pH 8, and irradiated 90s with UV light to mimic the in situ loading process.

	M	N	R^2
pH8	2,88E-03	-1,77E-05	>0.99
pH6	2,25E-02	-5,66E-05	>0.99

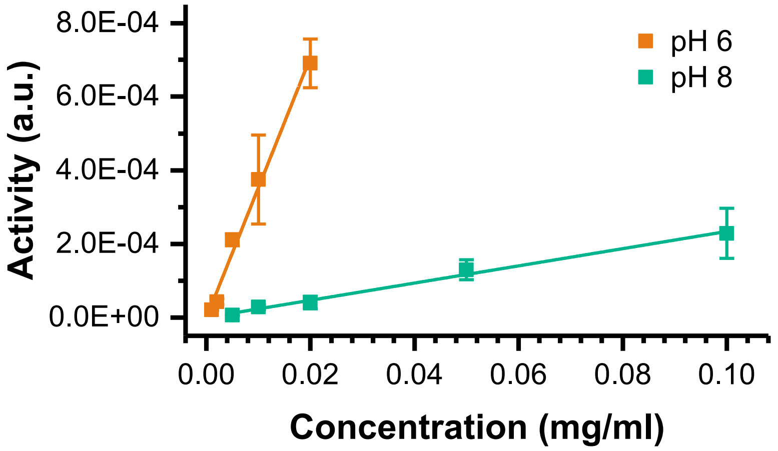

Figure S44: Calibration of Myoglobin activity, the enzyme is dissolved overnight at pH 6 and then brought back to basic conditions to mimic the the post loading process.

	M	N	R^2
pH8	2,34E-03	-1,16E-07	≈ 0.99
pH6	3,54E-02	-1,09E-06	≈ 0.99

It can be seen from the cyclic switching activity that, even though, the purification power was enhanced for Myoglobin the sample of the post approach still shows a slight activity at pH 8 during the beginning of the cyclic experiments. This might be explained by some residual

myoglobin that is still adsorbed at location 1 and is therefore also accessible for the substrates at pH 8.

In order to calculate loading efficiencies from enzyme activities we calibrated the enzyme activity by the mass concentration of the enzymes. It should be noted that storage at different pH values as well as the irradiation with UV light lead to certain decrease of activity. In order to compensate for that the calibration with known concentrations was treated like polymersome samples, e.g. stirring at pH 8 to 9 for four days with subsequent 90 s of UV irradiation to exactly replicate the conditions of in situ loading.

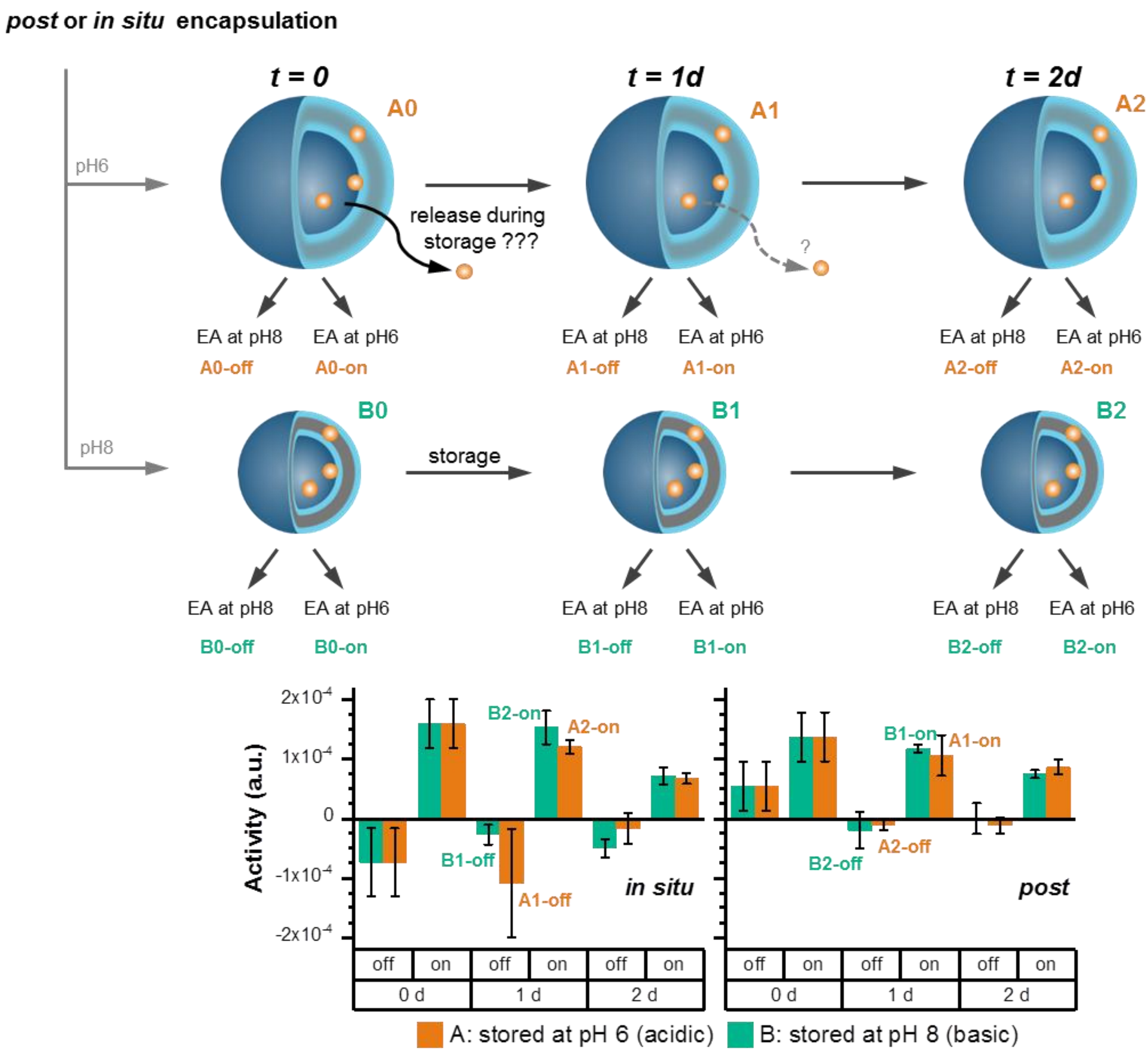

Figure S45: Myoglobin encapsulated in polymersomes; schematic outline of investigations concerning enzyme release during storage at different pH over 2 days. EA = enzyme activity assay.

Visually, the enzymatic activity of both, post and in situ loaded samples is very similar. It should be noted however, that the activity of myoglobin prepared according to the treatment conditions of post-loading shows generally slightly lower activity than the enzyme after the preparation at in-situ conditions.

4 Supplementary References

(1) Gumz, H.; Lai, T. H.; Voit, B.; Appelhans, D. Fine-Tuning the pH Response of Polymersomes for Mimicking and Controlling the Cell Membrane Functionality. Polym. Chem. **2017**, 8 (19), 2904–2908.

(2) Gaitzsch, J.; Appelhans, D.; Gräfe, D.; Schwille, P.; Voit, B. Photo-Crosslinked and pH Sensitive Polymersomes for Triggering the Loading and Release of Cargo. Chem. Commun. **2011**, 47 (12), 3466–3468.

(3) Iyisan, B.; Kluge, J.; Formanek, P.; Voit, B.; Appelhans, D. Multifunctional and Dual-Responsive Polymersomes as Robust Nanocontainers: Design, Formation by Sequential Post-Conjugations, and PH-Controlled Drug Release. Chem. Mater. **2016**, 28 (5), 1513–1525.

(4) Appelhans, D.; Komber, H.; Abdul Quadir, M.; Loos, A.; Schwarz, S.; Richter, S.; Aigner, A.; Müller, M.; Haag, R.; Voit, B.: Hyperbranched PEI with various oligosaccharide architectures: Synthesis, characterization, ATP complexation and cellular uptake. Biomacromolecules **2009**, 10, 1114-1124.

(5) Gräfe, D.; Gaitzsch, J.; Appelhans, D.; Voit, B. Cross-Linked Polymersomes as Nanoreactors for Controlled and Stabilized Single and Cascade Enzymatic Reactions. Nanoscale **2014**, 6 (18), 10752–10761.

(6) Dobberstein, P.; Schroeder, E. Accurate Mass Determination of a High Molecular Weight Protein Using Electrospray Lonization with a Magnetic Sector Instrument. Rapid Commun. Mass Spectrom. **1993**, 7 (9), 861–864.

(7) Heymann, E.; Junge, W. Characterization of the Isoenzymes of Pig-Liver Esterase 1. Chemical Studies. Eur. J. Biochem. **1979**, 95 (3), 509–518.

(8) Kilcawley, K. N.; Wilkinson, M. G.; Fox, P. F. Determination of Key Enzyme Activities in Commercial Peptidase and Lipase Preparations from Microbial or Animal Sources. Enzyme Microb. Technol. **2002**, 31 (3), 310–320.

(9) Charney, J.; Tomarelli, R. M. A Colorimetric Method for the Determination of the Proteolytic Activity of Duodenal Juice. J. Biol. Chem. **1947**, 171 (2), 501–505.
